# Fecal microbiome of patients with ulcerative colitis reflects their phenotype and inflammatory level

**DOI:** 10.1038/s41598-026-44895-6

**Published:** 2026-05-19

**Authors:** Nicolas Maziers, Emmanuelle Le Chatelier, Florian Plaza Oñate, Sébastien Fromentin, Florence Thirion, Nicolas Pons, Natalia Borruel, Francesc Casellas, Antonio Torrejon, Virginia Robles-Alonso, Chaysavanh Manichanh, Encarna Varela, Muriel Derrien, Patrick Veiga, Raish Oozeer, Shinichi Sunagawa, Vincent Lombard, Nicolas Terrapon, Bernard Henrissat, the MetaHIT consortium, Mathieu Almeida, Maria Antolin, François Artiguenave, Manimozhiyan Arumugam, Jean-Michel Batto, Marcelo Bertalan, Hervé Blottière, Peer Bork, Christian Brechot, Thomas Bruls, Søren Brunak, Kristoffer S. Burgdorf, Paul Igor Costea, Antonella Cultrone, Willem M. de Vos, Christine Delorme, Gérard Denariaz, Rozenn Dervyn, Doré Joël, Gwen Falony, Qiang Feng, Kristoffer Forslund, Niels Grapup, Eric Guedon, Valborg Gudmundsdottir, Florence Haimet, Torben Hansen, Rajna Hercog, Falk Hildebrand, Alexandre Jamet, Torben Jørgensen, Agnieska S. Junker, Catherine Juste, Ghalia Khaci, Sean Kennedy, Michiel Kleerebezem, Jan Knoll, Karsten Kristiansen, Jens Roat Kultima, Séverine Layec, Denis Le Paslier, Marion Leclerc, Pierre Leonard, Florence Levenez, Junhua Li, Christine M’Rini, Emmanuelle Maguin, Daniel R. Mende, Alexandre Mérieux, Trine Nielsen, Henrik Bjørn Nielsen, Julian Parkhill, Helle Krogh Pedersen, Oluf Pedersen, Eric Pelletier, Edi Prifti, Junjie Qin, Jeroen Raes, Simon Rasmussen, Pierre Renault, Maria Rescigno, Nicolas Sanchez, Thomas Sicheritz-Ponten, Julien Tap, Sebastian Tims, Keith Turner, Maarten van de Guchte, Johan E. T. van Hylckama Vlieg, Gaetana Vandemeulebrouck, Henrik Vestergaard, Sara Vieira-Silva, Anita Yvonne Voigt, Jian Wang, Jun Wang, Jean Weissenbach, Yohanan Winogradski, Takuji Yamada, Huanming Yang, Erwin G. Zoetendal, Francisco Guarner, S. Dusko Ehrlich

**Affiliations:** 1 Université Paris-Saclay, INRAE, MGP, 78350 Jouy en Josas, France; 2 Intensive Care Unit, Hospital Center Sud Francilien, 91100 Corbeil-Essonnes, France; 3 Digestive System Research Unit, Vall de Hebron Institute of Research (VHIR), Ciberehd, 08035 Barcelona, Spain; 4 Danone Nutricia Research, Palaiseau, France; 5 European Molecular Biology Laboratory, Structural and Computational Biology Unit, 69117 Heidelberg, Germany; 6 Department of Biology and Swiss Institute of Bioinformatics, University of Zurich, 8057 Zurich, Switzerland; 7 Architecture et Fonction des Macromolécules Biologiques (AFMB), CNRS, Aix Marseille Université, Marseille, France; 8 INRAE, USC1408 Architecture et Fonction Des Macromolécules Biologiques (AFMB), Marseille, France; 9 Department of Biotechnology and Biomedicine (DUT Bioengineering), Technical University of Denmark, Lynglby, Denmark; 10 Centre for Host-Microbiome Interactions, Dental Institute Central Office, Guy’s Hospital, King’s College London, London, SE1 9RT UK; 11 Department of Clinical and Movement Neurosciences, UCL Queen Square Institute of Neurology, Queen Square, London, WC1N 3RX UK; 12 Institut National de la Recherche Agronomique, US1367 Metagenopolis, 78350 Jouy en Josas, France; 13 Institut National de La Recherche Agronomique, UMR14121 MICALIS, 78350 Jouy en Josas, France; 14 Digestive System Research Unit, University Hospital Vall d’Hebron, Ciberehd, 08035 Barcelona, Spain; 15 Commissariat à l’Energie Atomique, Genoscope, 91000 Evry, France; 16 Centre National de la Recherche Scientifique, UMR8030, 91000 Evry-Courcouronnes, France; 17 Université d’Evry Val d’Essone, 91000 Evry, France; 18 European Molecular Biology Laboratory, Meyerhofstrasse 1, 69117 Heidelberg, Germany; 19 Center for Biological Sequence Analysis & Novo Nordisk Foundation Center for Biosustainability, Technical University of Denmark, 2800 Kongens Lyngby, Denmark; 20 Molecular Medicine Partnership Unit, University of Heidelberg and European Molecular Biology Laboratory, 69120 Heidelberg, Germany; 21 Max Delbrück Centre for Molecular Medicine, 13125 Berlin, Germany; 22 Institut Mérieux, 17 Rue Burgelat, 69002 Lyon, France; 23 Department of Systems Biology, Center for Biological Sequence Analysis, Technical University of Denmark, 2800 Kongens Lyngby, Denmark; 24 Novo Nordisk Foundation Center for Protein Research, Disease Systems Biology, Faculty of Health and Medical Sciences, University of Copenhagen, 2200 Copenhagen, Denmark; 25 Hagedorn Research Institute, Genteofte, Denmark; 26 Laboratory of Microbiology, Wageningen University, Wageningen, The Netherlands; 27 Department of Bacteriology and Immunology, University of Helsinki, N00014 Helsinki, Finland; 28 Danone Research, 91120 Palaiseau, France; 29 VIB Center for the Biology of Disease, Katholieke Universiteit Leuven, 3000 Leuven, Belgium; 30 Department of Microbiology and Immunology, Laboratory of Molecular Bacteriology, Rega Institute for Medical Research, Katholieke Universiteit Leuven, 3000 Leuven, Belgium; 31 Bejing Genomics Institute (BGI)-Shenzhen, Shenzhen, 518083 China; 32 Department of Biology, University of Copenhagen, 2100 Copenhagen, Denmark; 33 The Novo Nordisk Foundation Center for Basic Metabolic Research, Faculty of Health and Medical Sciences, University of Copenhagen, 2200 Copenhagen, Denmark; 34 Faculty of Health Sciences, University of Southern Denmark, 5000 Odense, Denmark; 35 Department of Bioscience Engineering, Vrije Universiteit Brussel, 1040 Brussels, Belgium; 36 Research Centre for Prevention and Health, Capital Region of Denmark, 2600 Glostrup, Denmark; 37 Department of Public Health, Faculty of Health and Medical Sciences, University of Copenhagen, 2600 Copenhagen, Denmark; 38 Faculty of Medicine, University of Aalborg, 9100 Aalborg, Denmark; 39 Gut Biology & Microbiology, Danone Research, Centre for Specialized Nutrition, Wageningen, The Netherlands; 40 BGI Hong Kong Research Institute, Hong Kong, China; 41 School of Bioscience and Biotechnology, South China University of Technology, Guangzhou, China; 42 Daniel K Inouye Center for Microbial Oceanography Research and Education (C-MORE), University of Hawaii at Manoa, Honolulu, HI USA; 43 The Wellcome Trust Sanger Institute, Hinxton, Cambridge, CB101SA UK; 44 Hagedorn Research Institute, 2820 Gentofte, Denmark; 45 Institute of Biomedical Science, Faculty of Health and Medical Sciences, University of Copenhagen, 2200 Copenhagen, Denmark; 46 Institute of Cardiometabolism and Nutrition, 75013 Paris, France; 47 IEO - Istituto Europeo Di Oncologia, Via Giuseppe Ripamonti, 435, 20141 Milano, Italy; 48 Department of Applied Tumor Biology, Institute of Pathology, University Hospital Heidelberg, 69120 Heidelberg, Germany; 49 James D. Watson Institute of Genome Science, Hangzhou, China; 50 Princess Al-Jawhara Al Brahim Centre of Excellence in Research of Hereditary Disorders (PACER-HD), Faculty of Medicine, KAU, Jeddah, Saudi Arabia; 51 Department of Biological Information, Tokyo Institute of Technology, Yokohama, Japan

**Keywords:** Shotgun, Metagenomic species, Richness, Enterotypes, Relapses, Inflammation, Diseases, Gastroenterology, Microbiology

## Abstract

Inflammatory bowel diseases affect ever-increasing numbers of individuals worldwide. Alterations of the intestinal microbiome were reported for Crohn’s disease and at relapse in Ulcerative Colitis (UC); they were not clearly detected in UC at remission. Here we report the characterization of the microbiome by quantitative metagenomics in a cohort of 121 individuals, composed of 65 UC adult patients in remission and 56 healthy controls. A cross-sectional comparison revealed substantial microbiome differences, patients in remission having lower microbiome richness and paucity of the Ruminococcus species driven enterotype. The observed microbiome alterations allowed robust classification of patients by intestinal species abundance, yielding an area under the curve (AUC) of 0.87 in a Receiver-Operator Characteristic (ROC) analysis. Loss of richness was linked to an aggressive UC phenotype and to the importance of past relapses; it was associated with a worse IBD quality of life score (IBDQ-36). Unexpectedly, onset of inflammatory bouts, as assessed by white blood cell count and fecal calprotectin levels, was associated with higher richness; in a longitudinal study of patients at high risk of disease flare, we observed a link between increasing gut microbiome richness over time and calprotectin level, in turn related to clinical inflammatory response and relapse.

**Supplementary Information:**

The online version contains supplementary material available at 10.1038/s41598-026-44895-6.

## Introduction

A dramatic increase in the incidence of UC has been observed over the past decades, first in developed countries but then also in developing regions. The increased incidence has paralleled changes in diet, sanitation conditions, and lifestyle habits^1,2^. Because mortality is low and the disease is most often diagnosed in the young, data suggest that the global prevalence will continue to increase, as well as the social and public health burden.

Crohn’s disease and UC are two types of inflammatory bowel disease (IBD.) Phenotypically, they are distinct^3^ in terms of anatomical-pathological features and clinical manifestations, and their genetic background is not identical. Several UC-associated loci have been identified^4^, most of them being implicated in signaling pathways of innate immunity or intestinal barrier function. However, most individuals with genetic susceptibility do not develop the disease indicating that environmental factors are necessary contributors to the pathogenesis of UC, and primarily responsible for its growing incidence around the globe.

There is considerable clinical and experimental evidence supporting the concept that alterations of the gut microbial ecosystem contribute to development of IBD^3,5–8^. Microbiome alterations have been consistently recognized in Crohn’s disease and confirmed in a large cohort of new-onset patients naïve to medical therapy^9^. In UC, studies have shown decreased bacterial diversity in the gut microbiota with expansion of putative deleterious groups (Proteobacteria) combined with a decrease in so-called protective groups (Faecalibacterium, Lachnospiraceae, Roseburia)^10^. However, these changes were mostly described in patients suffering from clinical relapse or whose UC disease activity status was not clearly stated at the time of sampling.

The aim of this observational study was to investigate microbiome alterations in UC patients during remission as compared with healthy subjects. We enrolled two cohorts of patients differing by their disease intensity profile (time on remission and rate of past of relapses) and examined them cross-sectionally and longitudinally. The first UC cohort (high risk of relapse) was followed for one year or until clinical relapse to identify microbial changes associated with disease flare while the second UC cohort (“mild” disease) was part of a 12-week double blind, randomized placebo-controlled study exploring the effect of probiotics contained in a fermented milk product on the quality of life of UC patients and their fecal microbiome. We employed our quantitative methodology^11^ based on the integrated gene catalog^12^, structured in metagenomic species (MGS)^13,14^, to conduct an analysis of sequenced fecal DNA, both in term of ecosystem’s proxies, namely richness^15^ or enterotypes^16,17^, and as taxonomic entities with their functional potential: all through a fully exploratory data driven approach focused on UC patient clinical characteristics.

## Results

### Alterations of the microbiome in UC remission

**Fig. 1:**
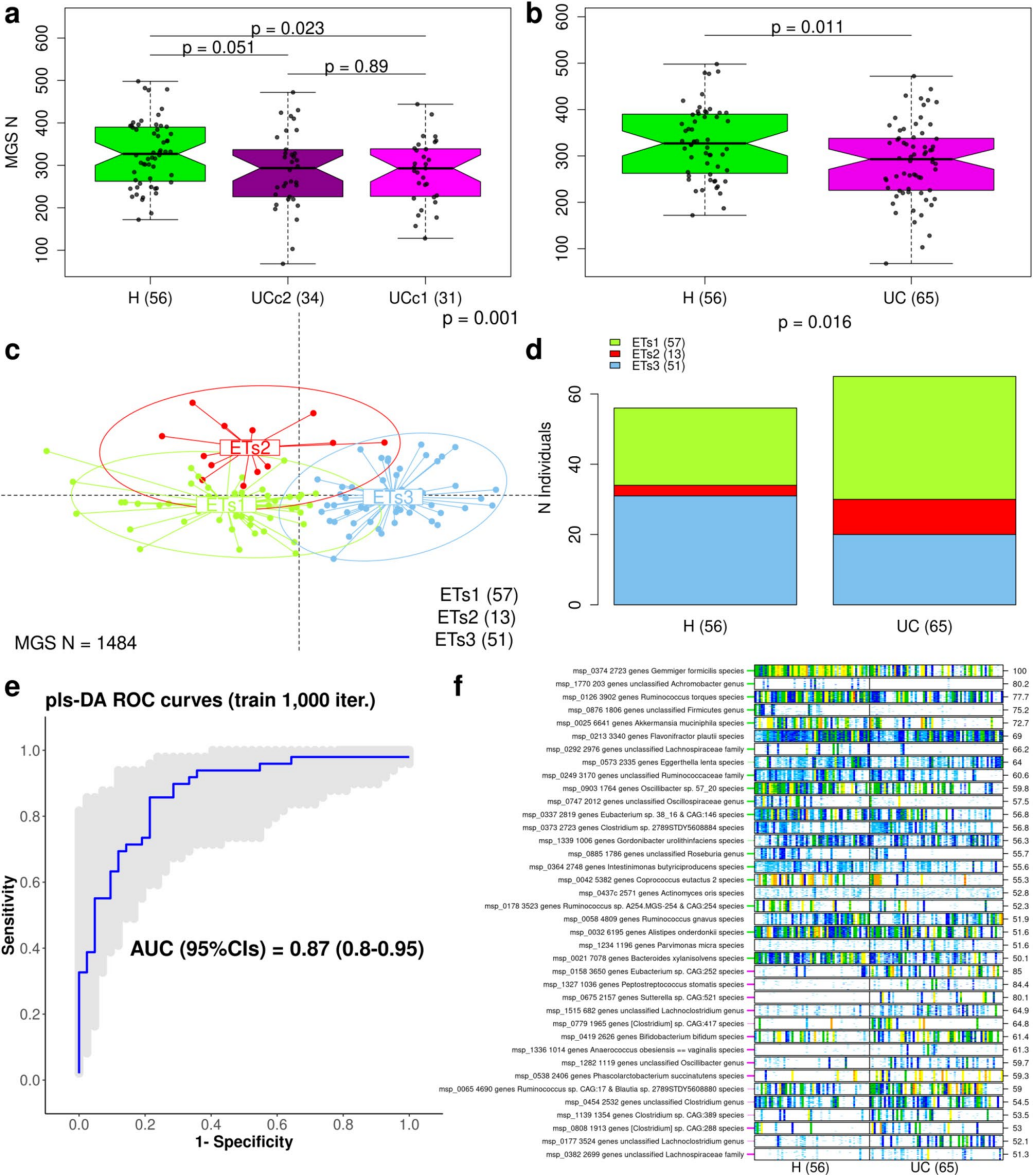
Microbiome differences between H subjects and UC patients. (**a**) Number of MGS in healthy (H) cohort, ulcerative colitis cohort 2 (UCc2) and UC cohort 1 (UCc1) subjects (n in parentheses). Significance of differences was assessed by the Wilcoxon rank sum test for each comparison, the respective p-values are indicated. (**b**) Number of MGS in H and pooled UC cohorts. Probability was computed by Wilcoxon rank sum test. (**c**) Between-class analysis (bca) of principal coordinate analysis (PCoA) based on Bray Curtis distances calculated from the weighted relative abundances of 1484 MGS detected in 121 individuals (H and UC). The first principal plan represented 21.6% and 7.0% of whole inertia for first (horizontal) and second (vertical) axes, respectively. The between-class inertia percentage was 15.9%. Enterotypes species (ETs, see Methods) are displayed. Probability was computed by Monte-Carlo permutation test on the between-class inertia. (**d**) ETs of H and UC subjects. Probability was computed by a Fisher’s exact test. (**e**) Receiver operating characteristic (ROC) analysis of partial least square discriminant analysis (pls-DA) on the train dataset (Methods) based on weighted MGS relative abundances for classification of H vs UC patients. Each of the 1,000 iterations shown in grey, the blue curve is closest to the median area under the curve (AUC). Lower and upper bounds correspond to the medians of the 95% confidence intervals (95% CIs) for 1,000 iterations. (**f**) Abundance heatmap of the MGS most often used for pls-DA based classification of H and UC subjects. For each clinical status, MGS with highest variable importance (varImp, median over 1,000 iterations) are shown (median varImp value displayed as right-hand label). MGS taxonomic assignment and gene number is on the left. Green and magenta ticks following the assignment denote significant enrichment in H and UC cohort, respectively. Thin and thick lines indicate significant occurrence difference (Fisher exact test) only or of both occurrence and abundance (Wilcoxon rank sum test) difference, respectively. Colours indicate the abundance of 50 marker genes (white: not detected; increasing abundance from blue to red). Individuals are ordered by decreasing richness, separately for H and UC cohorts.

UC cohort 1 (UCc1) included 31 UC patients in clinical remission and stable medication for at least 3 months, but highly active disease in the past (> 3 relapses during the past 5 years). UC cohort 2 (UCc2) included 34 UC patients in clinical remission and stable medication for at least one year and mild activity in the previous 5 years (< 1 relapse/year). Healthy cohort (H) included 56 healthy individuals (see study diagram, Extended Data Fig. 1). We compared the number of metagenomics species^13,14^ (MGS) in fecal samples obtained at entry from all individuals (Fig. 1a). Species count per sample, named hereafter richness^13,15^, was significantly lower in UCc1 (n = 31; p = 0.023) or almost so in UCc2 (n = 34; p = 0.051) than in H (n = 56). Clinical variables other than those related to the above-mentioned enrolment criteria were not significantly different between UCc1 and UCc2 (Supplementary Table 1); we therefore pooled the two cohorts. A significant 10% richness difference between healthy subjects (n = 56; median 327 MGS), and UC patients (n = 65; median 293 MGS) was found (p = 0.011; Fig. 1b). As the H group was younger than the UC one (p = 0.004; Supplementary Table 2) we adjusted richness for age as co-variable and found again a significant decrease in patients (linear model adjusted p = 0.009).

We also used enterotyping^16^ to capture the architecture of fecal microbiome of our full cohort (n = 121). We computed enterotypes from MGS abundances by the Dirichlet multinomial mixtures model approach^17^. Three clusters were obtained (Fig. 1c), named enterotypes species (ETs): ETs1 is largely driven by *Bacteroides uniformis* or *Bacteroides vulgatus* (n = 57), ETs2 by *Prevotella copri* phylogroup 1 (n = 13) and ETs3 by *Ruminococcus* sp. JC304 (n = 51, Extended Data Fig. 2a-d). ETs were associated (p = 0.016) with the health status (H or UC, Fig. 1d). The commonly used genus abundance based enterotyping also delimited 3 clusters (Extended Data Fig. 2e), or “classic” enterotypes (ET) with their genus drivers, as in^18^. ET and ETs were clearly related (p = 8.1 × 10^–20^; Extended Data Fig. 2f). However, in contrast to ETs, there was no significant ET dependence (p = 0.28) on the health status (Extended Data Fig. 2g).

**Fig. 2:**
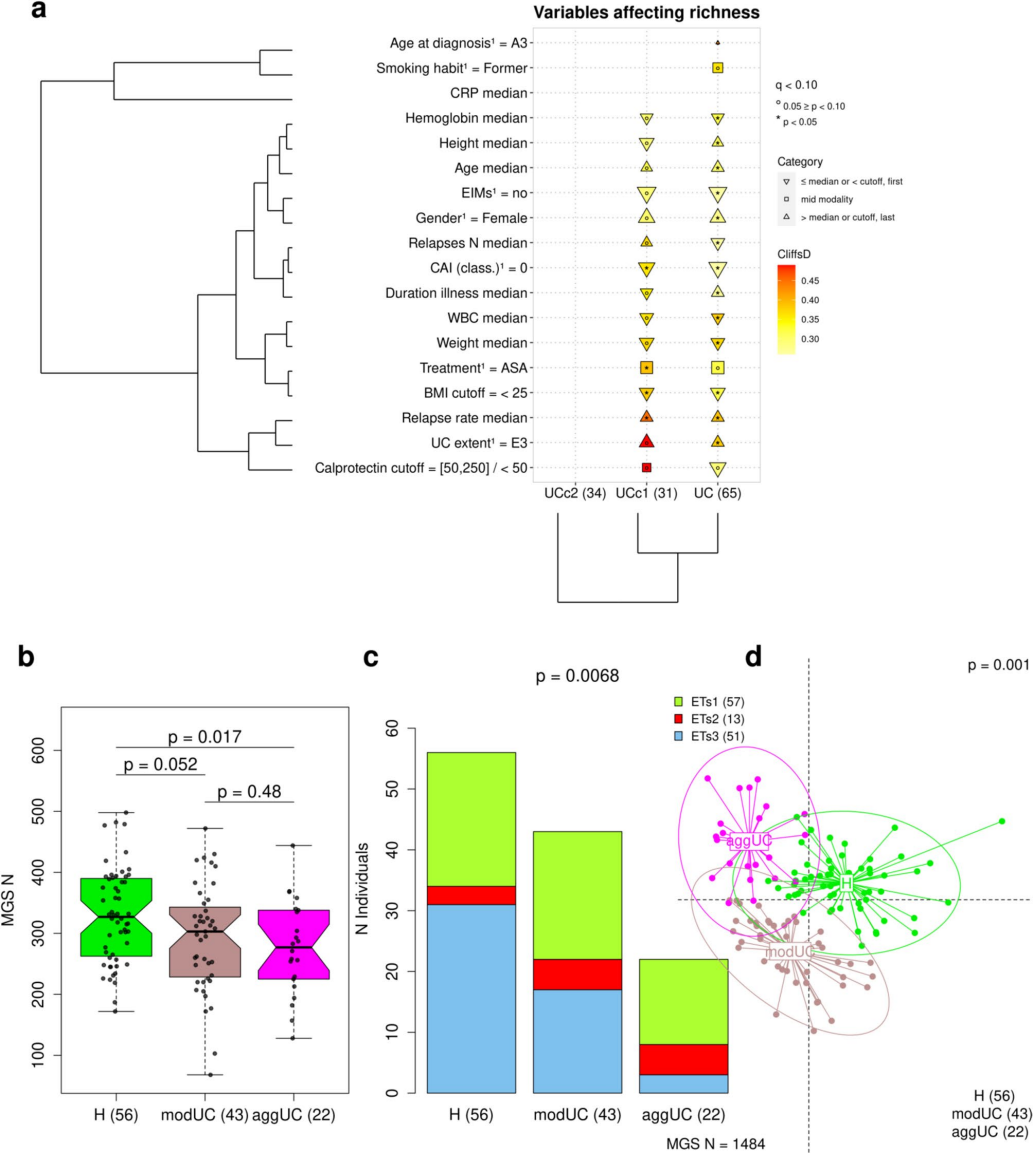
Microbiome in link with clinical characteristics to delimit UC subgroups. (**a**) Effect size heatmap of richness reduction in UC cohorts, compared with H, according to their most relevant clinical categorized variables. Cohorts (bottom) were: UCc2, UCc1 or pooled (UC) patients (n). Variables (left) were from Supplementary Table 1 as such (^1^) or were discretized (Methods), based on their median (inferior/equal ≤ ; strictly superior >) or on cut-off (strictly inferior < ; >) values (BMI < or > 25 kg/m^2^; fecal calprotectin < 50 or [50,250] or > 250 µg/g). Confronted with H (n = 56), richness was compared for each variable (k = 2 or k = 3 categories) from each patient cohort. Kruskal–Wallis rank sum test was computed (p-values and Benjamini–Hochberg q-values) with pinned icons if q < 0.10 and on these, tagged (°) if 0.10 > p ≥ 0.05 or (*) if p ≤ 0.05 (top right). Cliff’s Delta (CliffsD) was calculated for the category with the lowest probability from (with H) two-by-two Wilcoxon rank sum test (Supplementary Table 3). Categories (middle right) displayed as icons whose size are = n_category/(n_cohort/k) and shapes are: (∇) if it is ≤ median or < cutoff or first ordered modality, (□ k = 3) if is mid or intermediate level if cutoff, and (∆) if it is > median or > cutoff or last ordered modality. If required, some of them are on left specified ( =). CliffsD are, inside icons, colour-coded (light yellow if small, dark yellow-orange if medium, red if large) and expressed as dendrograms (Ward’s method on Euclidian distance) of cohorts and variables. (**b**) Detected MGS in H, moderate UC (modUC) and aggressive UC (aggUC) subgroups (n); Wilcoxon rank sum tests. (**c**) Enterotypes species of H, modUC and aggUC subgroups; Fisher’s exact test. (**d**) Between-class analysis of principal component analysis (PCA) calculated from the weighted abundance of 1484 MGS in the 121 individuals. The first axis represented 7.3% (horizontal) and second axis 3.6% (vertical) of whole inertia. The between-class inertia was 2.5%, p-value resulted from Monte-Carlo permutation test done on this inertia. H, modUC and aggUC subgroups indicated.

Species-level differences between healthy subjects (n = 56) and UC patients (n = 65) were explored to construct a classification model relying solely on MGS relative abundances. After having compared a selection of tools from statistical and machine learning class (Extended Data Fig. 3 and Methods), we deployed partial least square discriminant analysis (pls-DA) to model our data. Applying this classifier 1,000 times, the median of fitted models yielded an area under the curve in the ROC analysis of 0.87 and 0.88 on the training and testing data sets, respectively (Fig. 1e). The species most important for the discrimination, assessed by the number of models that used them, are displayed in Fig. 1f. As recently published by Zheng et al^8^, among the species depleted in patients were short chain fatty acid producers like *Gemmiger formicilis*, phylogenetically close to *Faecalibacterium*, *Roseburia* species, *B. xylanisolvens,* and the mucin degraders like *Ruminococcus torques* and *Akkermansia muciniphila*. Oral microbiome species (*Peptostreptococcus stomatis*) and pathobionts such as *Sutterella* and *Anaerocccus* species were enriched in patients.

### UC subgroups according to clinical characteristics and microbiome

Links between microbiome and clinical characteristics of patients were scrutinized adopting a strategy starting with variables discretization (Methods and Supplementary Table 1) across either the pooled UC cohort or the two initial cohort parts (UCc1, UCc2), focusing on microbial richness difference compared to H cohort (Fig. 2a and Supplementary Table 3). While no variables were found to affect richness in the UCc2 cohort (q > 0.1), in UCc1, three distinct clinical categories had a larger effect size: pancolitis (E3 UC extent from Montreal classification), higher rate of past relapses (> 1.1/year) and an intermediate level of fecal calprotectin ([50,250] µg/g) as shown in Extended Data Fig. 4a–c.

Patients with intermediary calprotectin level had a high relapse rate and were more frequent among the Montreal E2 and E3 than the E1 category (Extended Data Fig. 4d,e). These observations led us to group the UCc1 patients with extended colitis (E2 and E3) into a separate phenotypic sub-cohort, termed aggressive UC (aggUC, n = 22). Conversely, we grouped the limited proctitis E1 UCc1 with UCc2 patients, assuming they had a more moderate UC (modUC, n = 43) disease than aggUC subgroup. When comparing microbiome structure within the two patient subgroups relative to healthy individuals a gradient from healthy over modUC to aggUC was clearly observed (Fig. 2b,c). Particularly striking was the depletion of the *Ruminococcus*-driven enterotype species in aggUC. Though less prominent, similar relationship was present with initial cohorts (Extended data Fig. 4f). Principal component analysis (PCA) of the relative abundance of the species detected (1484 MGS) in all individuals (n = 121) convincingly separated our three phenotypic groups (Fig. 2d) better than the initial ones (Extended data Fig. 4g).

### Microbiome—phenotype—genotype associations in aggressive UC

**Fig. 3:**
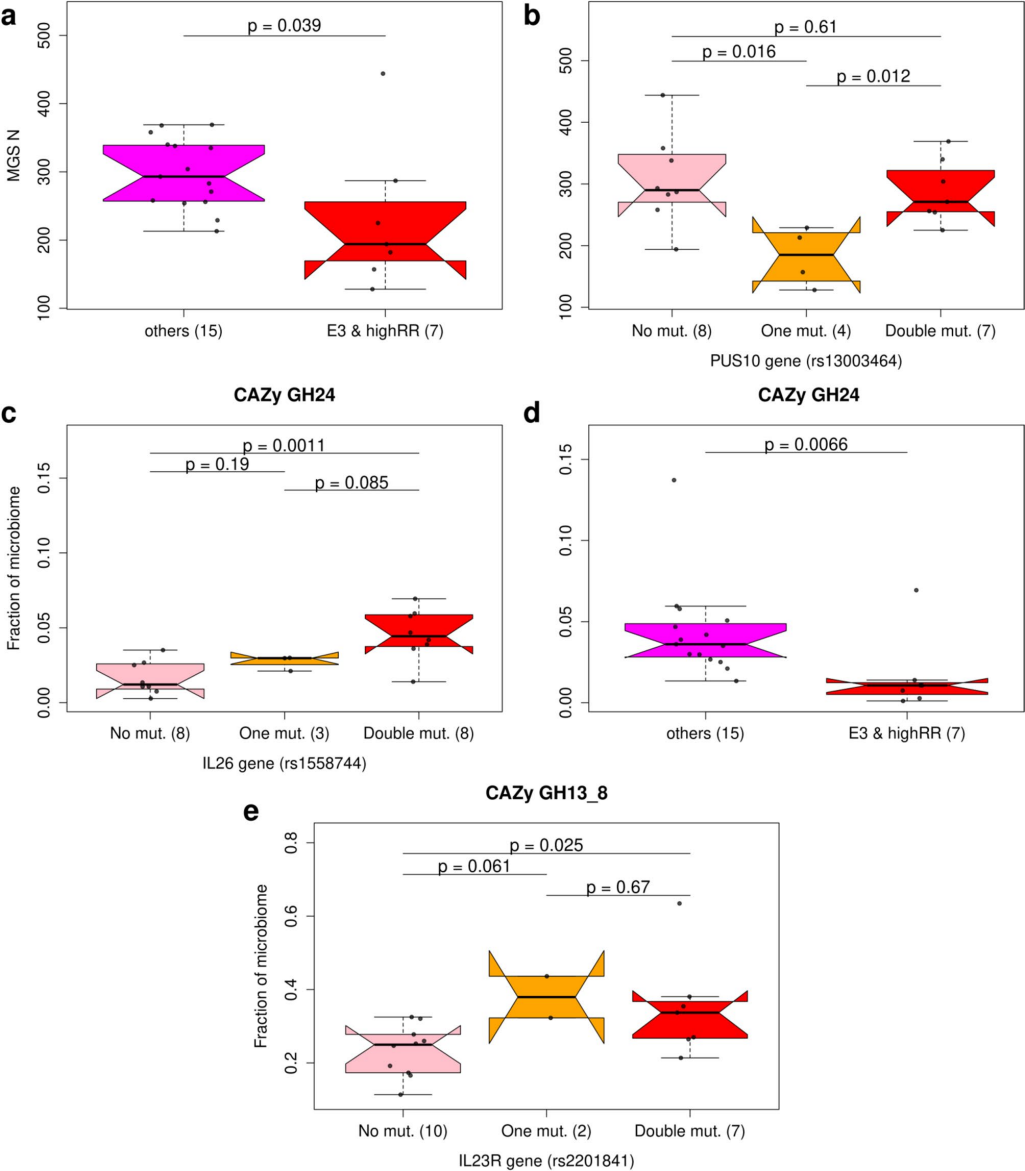
Microbiome—phenotype—genotype relation in aggressive UC subgroup (n = 22). (**a**) Number of MGS in patients being both E3 and high RR (n = 7) or not (n = 15); p-value was computed by Wilcoxon rank sum test. (**b**) Number of MGS in non-mutated (No mut.), single and double mutated allele (One mut. and Double mut., respectively) of the PUS10 gene (rs13003464). The p values were computed by a Wilcoxon rank sum test taken two by two. (**c**) Fraction of the microbiome encoding carbohydrate active enzymes (CAZy) of the glycoside hydroxylase family 24 (GH24) in IL26 mutants (rs1558744). Pairwise Wilcoxon rank sum test was performed. (**d**) Fraction of the microbiome encoding GH24 in patients being both E3 and high RR (n = 7) or not (n = 15); p-value was computed by Wilcoxon rank sum test. (**e**) Fraction of the microbiome encoding CAZy of the glycoside hydroxylase family 13, subfamily 8 (GH13_8) genes in function of the rs2201841 single nucleotide polymorphism (SNP) in IL23R gene. Wilcoxon rank sum test was applied.

The aggressive UC subgroup (aggUC, n = 22) corresponds to clinical profile marked by an aggressive course^19^, i.e. higher annually relapse rate prior to study, and anatomically extended disease. Remarkably, richness was dramatically decreased (Fig. 3a; p = 0.039 and Cliff’s Delta, CliffsD = 0.56) in patients (n = 7) with pancolitis (Montreal E3) and high relapse rate (annual past relapse rate > 1.1/year as median) compared to other aggUC (n = 15). Furthermore (Extended Data Fig. 5a–c), these 7 patients had also a significantly (p = 0.013) shorter duration of illness (median of 36 months), suggesting that they may be within the high-risk (or unstable) period of the first years of UC illness^20^. Besides phenotype, richness in aggUC seemed to follow host’s pseudouridine synthase 10, PUS10 gene at reference single nucleotide polymorphism (SNP), rs13003464 (reference SNP cluster or rs), from IBD genome wide associations studies^21^ with lower richness when one allele was mutated (Fig. 3b). The link between richness and this genetic susceptibility locus was not found (p = 0.80, Kruskal–Wallis rank sum test, data not shown) in healthy relatives (HR, n = 26) of UCc1 patients (n = 25) for whom genotyping had been performed (Methods). The association of this genotype with annual relapse rate and age at diagnosis (Montreal) (Extended Data Fig. 5d,e) categories suggested, as with the microbiome, a link between genotype and phenotype.

Unexpectedly (Fig. 3c), a connection appeared to be present between rs1558744, another UC risk SNP located in interleukin 26, IL26 gene, at the level of its allelic mutational status (p = 0.0011), and the fraction of microbiome encoding the genes of glycoside hydrolase family number 24 (GH24 of the carbohydrate active enzymes database {CAZy}; https://www.cazy.org/, Methods). Enzymes of this family correspond to viral lysozymes, sometimes integrated into bacterial genomes. We did not observe (p = 0.78, Kruskal–Wallis test, data not shown) this relation in HR (n = 26). Moreover, the level of this CAZy family was linked (p = 0.0066) with the E3-high relapse rate phenotype (Fig. 3d). In addition, this abundance of GH24 encoding genes was strongly correlated with richness (rho = 0.72, p = 0.00021) and ETs (Extended Data Fig. 5f,g). Similarly, CAZy GH family 13 subfamily 8 (GH13_8) genes abundance was associated to IL23 receptor, IL23R genetic polymorphism, namely rs2201841 (Fig. 3e). These enzymes have no known extracellular function; they are involved in bacterial glycogen metabolism where they catalyze the formation of α-1,6-branching points by transglycosylation. Once more, there was no equivalent link (p = 0.30, Kruskal–Wallis test, data not shown) in HR (n = 26). Notably (Extended Data Fig. 5h,i), the abundance of GH13_8 encoding genes was clearly anti-correlated with richness (rho = − 0.67, p = 0.00078) and associated with enterotype 2. Remarkably (Extended Data Fig. 5j,k), this abundance was also linked to fecal calprotectin 50–250 categorization and anti-correlated (rho = − 0.53, p = 0.011) with WBC.

### Microbiome links with subclinical inflammation in UC

**Fig. 4:**
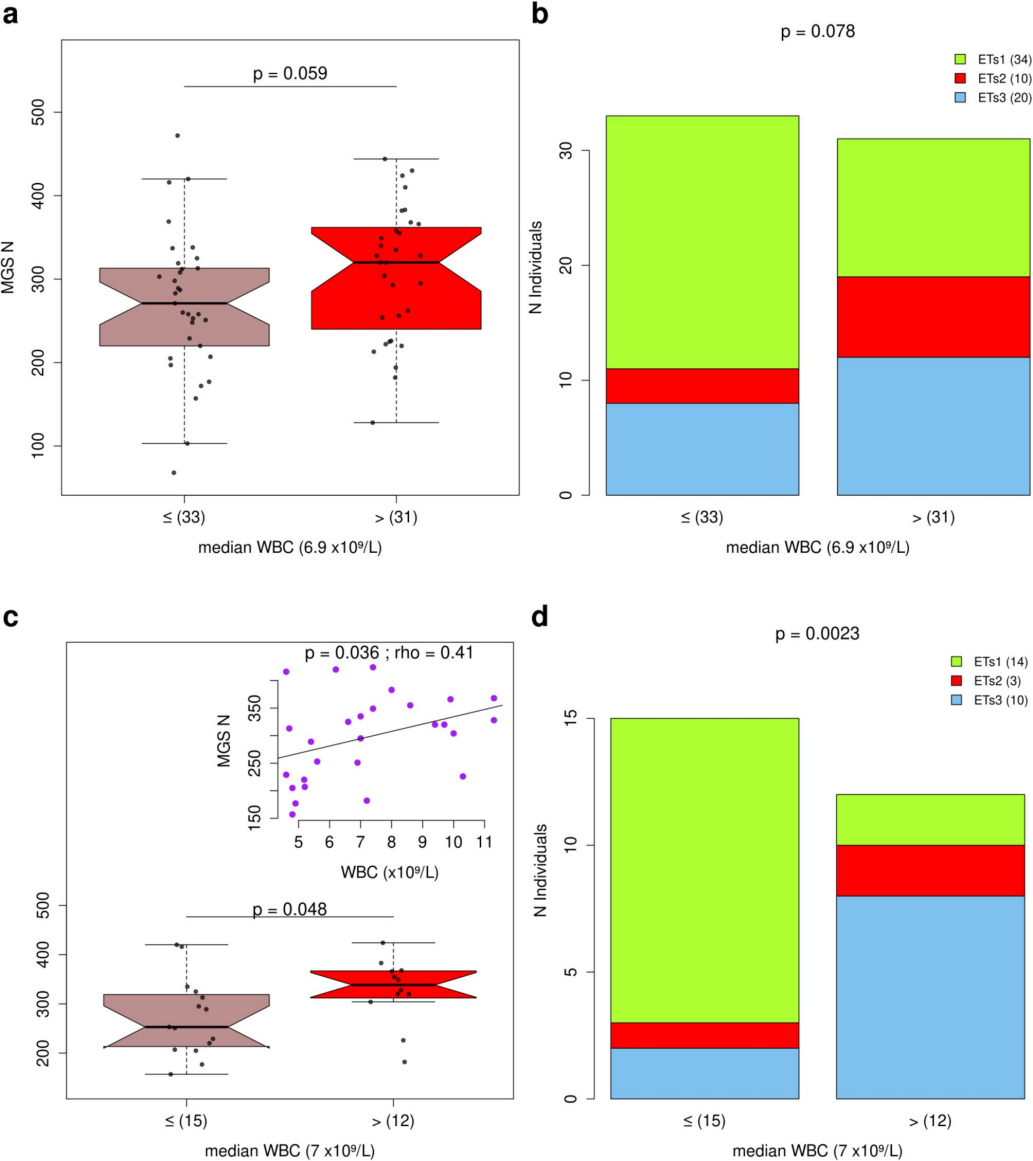
Relation between microbiome and subclinical inflammation in UC. a. Number of MGS in patients with white blood cells (WBC) count lower ( ≤) or higher ( >) than the median (6.9 × 10^9^/L); the pooled UC cohort was examined (p value was computed by a Wilcoxon rank sum test). (**b**) Enterotypes species (ETs) of patients WBC count lower or higher than the median. Probability was computed by a Fisher’s exact test. (**c**) Number of MGS in never smoker UC patients (n = 27) with WBC count lower or higher than the median (7 × 10^9^/L); p value was computed by a Wilcoxon rank sum test. Relation between WBC and number of species is shown as inset; Spearman’s rho correlation coefficient and the p value are indicated. (**d**) ETs of never smoker patients with WBC count lower or higher than the median. Probability was computed by a Fisher’s exact test.

We explored links between inflammatory markers (WBC and fecal calprotectin) and microbiome structure readouts (richness and ETs). Expectedly^22,23^, the two inflammation-related variables in pooled UC cohort (n = 65) were linked together but weakly (p = 0.054, Extended Data Fig. 6a). Surprisingly, patients in clinical remission with WBC above the median (6.9 × 10^9^/L) tended to have (p = 0.059, CliffsD = − 0.28) more species than those below (Fig. 4a); consistently, the two patient groups appeared to have different enterotype distribution (p = 0.078), *Ruminococcus*-driven enterotype (ETs3), which is associated with higher richness, being more frequent in the high WBC group (Fig. 4b).

Impact of smoking on WBC (Extended Data Fig. 6b) and on calprotectin (Extended Data Fig. 6c,d) could confound our observations. In this regard, we analyzed patients who never smoked (n = 27) and found the same positive connection between richness and discretized variable constructed on WBC (Fig. 4c; p = 0.048, CliffsD = − 0.46) to the point of being allowed to plot this positive correlation between richness and WBC (rho = 0.41, p = 0.036; inset Fig. 4c). Concordantly, richness-associated ETs3 was overrepresented in the high WBC group (Fig. 4d). Hence, in UC, richness appears to be associated with high WBC. Moreover, WBC and calprotectin were both linked to CAI classes (Extended Data Fig. 6e,f) showing a tendency to higher values of inflammation (p = 0.075 and p = 0.0056 respectively) in mildly symptomatic (CAI class 1–2) patients compared to the remaining asymptomatic (CAI 0) never-smoker UC patients. Taking them together, these observations suggest that high richness may not be necessarily favorable.

### Microbiome at flare

Twenty-five patients of the UCc1 cohort were included in a year-long longitudinal study (Extended Data Fig. 1). As anticipated^19^, 12 remained in remission (CAI < 4) while 13 relapsed (CAI ≥ 4). Relapse patients were sampled early at onset of flare-up symptoms before any therapeutic modification, and those on sustained remission at the end of follow-up (median time between sampling points was 6.1 and 12.8 months for patients that relapsed or not, respectively). While fecal calprotectin was significantly higher at the end of the study in relapse compared to remission patients (unpaired test, p = 0.0083; Extended Data Fig. 7a), WBC count of relapsers tended to decrease relative to their baseline value (paired test, p = 0.059; Extended Data Fig. 7b), possibly because of WBC migration into the inflamed mucosa.

**Fig. 5:**
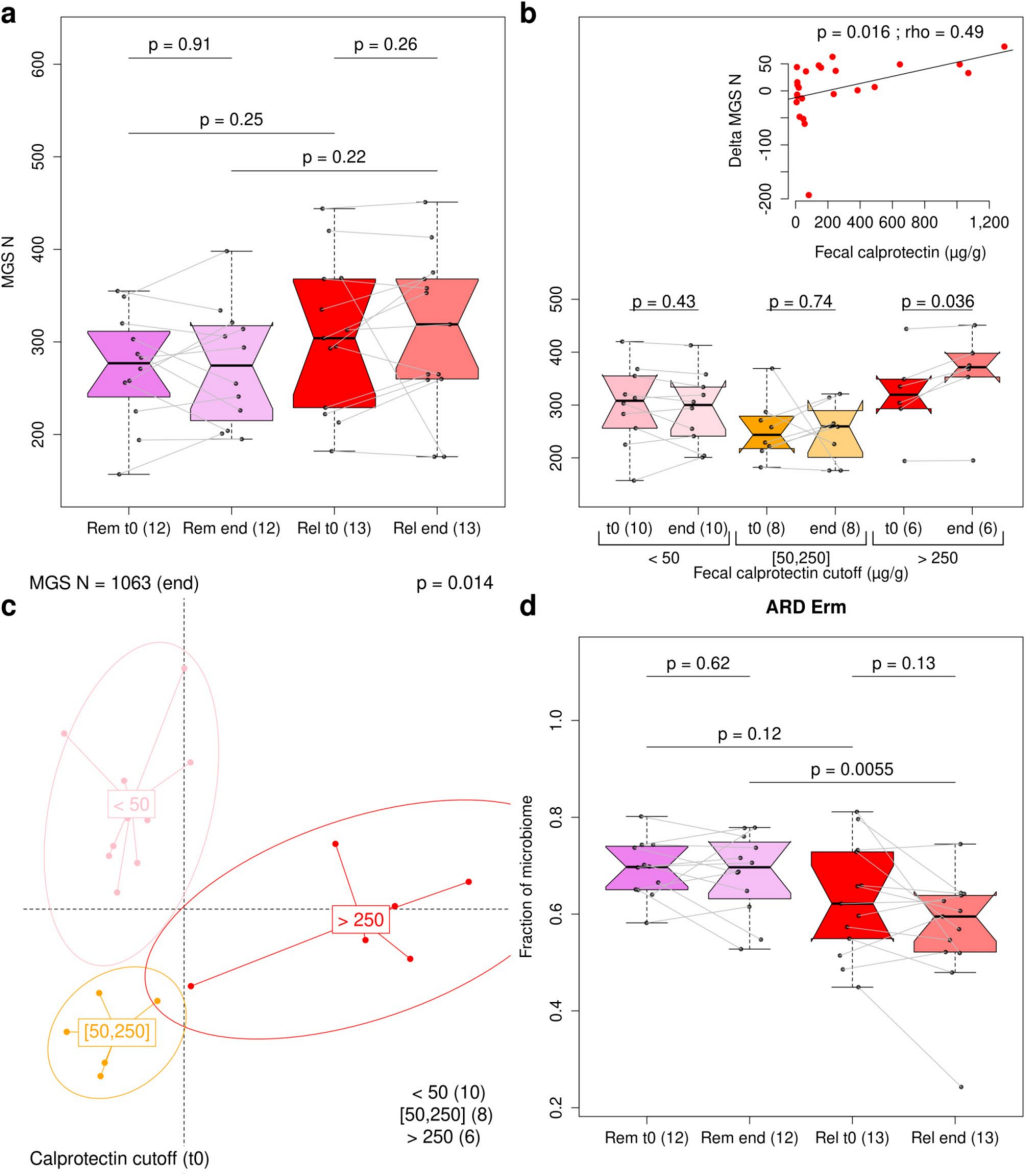
UC microbiome evolution during disease activity. (**a**) Number of MGS at baseline (t0) and end time points in 25 patients from UC cohort 1 time course, UCc1 TC; all were in remission at t0. About a half remained in remission (Rem, n = 12), others progressed to relapse (Rel, n = 13). Same individuals are connected by lines, p values were computed by pairwise paired or unpaired Wilcoxon tests (upper or lower values, respectively). (**b**) Number of MGS at t0 and end time points in the 25 patients in function of their fecal calprotectin levels at t0 (cutoff values of 50–250 µg/g were used). Same individuals are connected by lines; p values were computed by pairwise paired Wilcoxon tests. Relation between fecal calprotectin levels at t0 and richness difference between end and baseline (Delta) is shown in inset; Spearman’s rho correlation coefficient and the p value are indicated. (**c**) Between-class analysis (bca) of principal component analysis (PCA) calculated from the weighted relative abundances of 1063 MGS detected in the patients at endpoint. The first principal plan represented 11.3% and 9.3% of whole inertia for first (horizontal) and second (vertical) axes respectively. The between-class inertia percentage was 10.6%. Baseline calprotectin levels are indicated. The p value vas computed by a Monte-Carlo permutation test on the between-class inertia. (**d**) Fraction of the microbiome encoding antibiotic resistance determinant (ARD) erythromycin methyltransferase (Erm) family genes in patients remaining in remission and those that relapsed; p values were computed by pairwise paired or unpaired Wilcoxon tests (upper or lower values, respectively).

Of note, smoking habit impacted calprotectin levels at baseline, if dichotomized with 100 µg/g cutoff (Extended Data Fig. 7c), but not WBC (Extended Data Fig. 7d). Change in richness between baseline and end time point was not significant, both for remission and for relapse patients compared to themselves (paired tests, p = 0.91 and 0.26 respectively) or as a group (Fig. 5a). Interestingly, patients with highest calprotectin at baseline (> 250 µg/g; n = 6) increased significantly their richness (paired test, p = 0.036; Fig. 5b) and more globally, richness increase during time course was correlated to calprotectin at baseline (rho = 0.49, p = 0.016; inset Fig. 5b). Furthermore, the PCA analysis based on the relative abundance of the 1063 gut microbial species present at end point convincingly separated these UCc1 patients by their baseline calprotectin (based on 50–250 µg/g cut-off) category (p = 0.014, Fig. 5c), while the same analysis based on the 1060 MGS detected at baseline did not (p = 0.6, Extended Data Fig. 7e). Richness increase was evidently observed (paired test, p = 0.0067) in UCc1 patients with baseline calprotectin > 100 µg/g (n = 11) (Extended Data Fig. 7f,g). Former-smokers (n = 7) increased their richness during time course (paired test, p = 0.016; Extended Data Fig. 7h) in contrast to current (active) smokers (n = 6), who are overrepresented (p = 0.073; two-by-two Fisher’s exact test p = 0.048) in pauci-symptomatic (CAI 0 or CAI 1–2 classes) at end time point compared to more symptomatic (remission or not) never-smoker patients (n = 12; Extended Data Fig. 7i).

We also compared the fraction of species carrying antibiotic resistance determinants (ARD)^24^ (Methods) in patients that remained in remission (n = 12) or relapsed (n = 13) and observed (Fig. 5d) that the latter had significantly lower proportion (unpaired test, p = 0.0055) of species carrying the erythromycin methyltransferase (Erm) family genes. Coherently (Extended Data Fig. 7j), the fraction of the microbiome encoding Erm ARD was significantly higher (p = 0.0055) in individuals with lowest calprotectin levels at end point (< 50 µg/g, n = 5) compared to patients with the highest levels (> 250 µg/g; n = 11). In addition, that fraction was anti-correlated (rho = − 0.6, p = 0.0014) with Delta CAI (Extended Data Fig. 7k).

### Effects of fermented milk consumption on UC microbiome

**Fig. 6:**
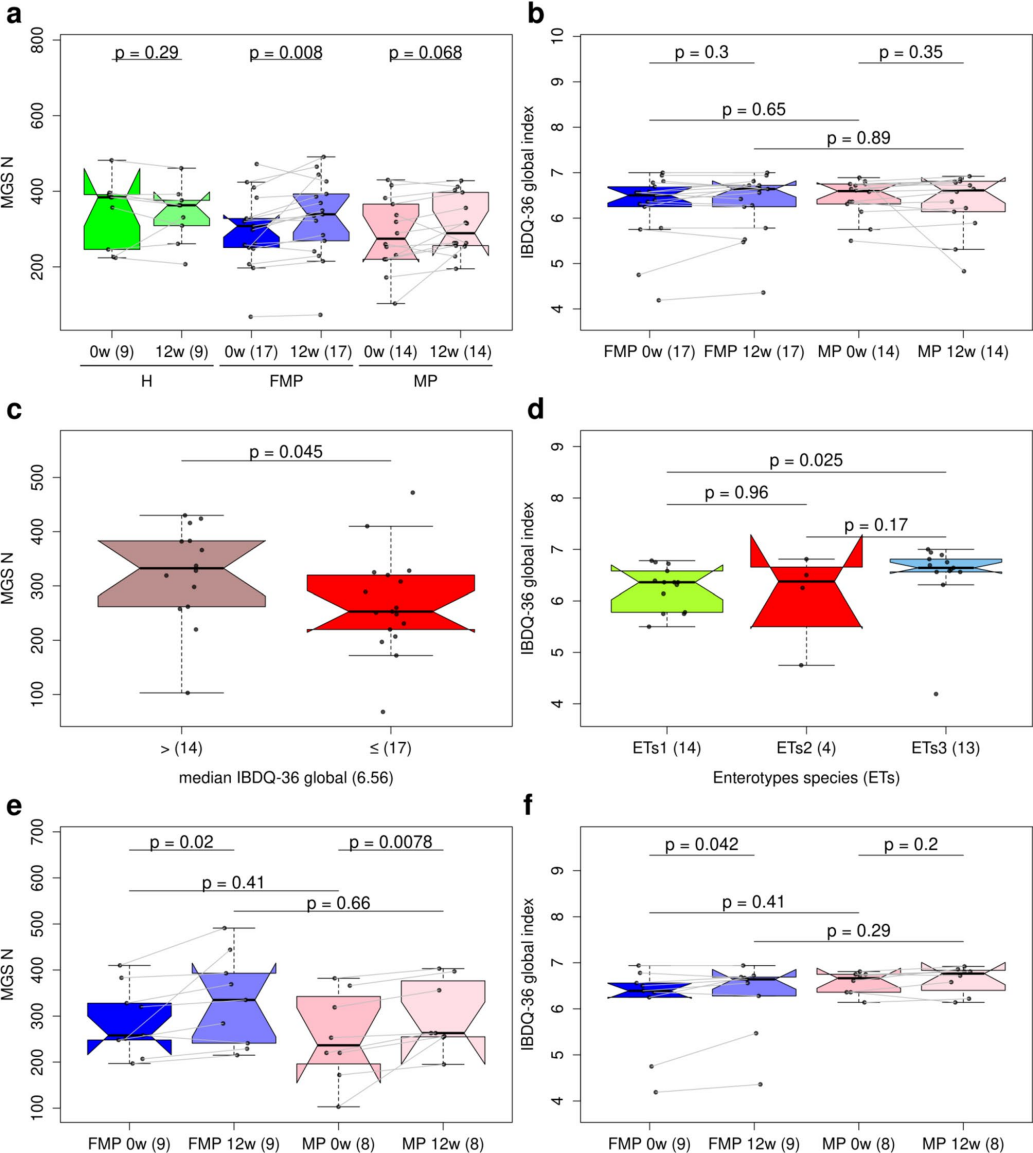
Effects of the consumption of a probiotic containing fermented milk product compared to control milk product. (**a**) Number of MGS at baseline (0 weeks; 0w) and end time points (12w) in 40 individuals. Of these, 9 were healthy (H), others were patients from the UC cohort 2, UCc2 trial; 17 consumed fermented milk product (FMP) and 14 acidified milk product (MP) for 12 weeks. Same individuals are connected by lines; the p values were computed by paired Wilcoxon tests. (**b**) Specific 36 items inflammatory bowel disease quality of life (IBDQ-36) questionnaire global index values at 0w and 12w in UCc2 patients consuming FMP or MP; p values were computed by pairwise paired or unpaired Wilcoxon tests (upper or lower values, respectively). (**c**) Baseline number of MGS in patients with IBDQ-36 score higher ( >) or lower ( ≤) than the median; p value was computed by a Wilcoxon rank sum test. (**d**) Baseline IBDQ-36 values of individuals assigned to different enterotypes species (ETs); p values were computed by pairwise Wilcoxon tests. (**e**) Number of MGS at baseline (0 weeks; 0w) and end time points (12w) in UCc2 patients, lacking *Akkermansia muciniphila* (akk-) and having fecal calprotectin < 250 µg/g (n = 17). Among them, 9 consumed FMP and 8 MP; p values were computed by pairwise paired or unpaired Wilcoxon tests (upper or lower values, respectively). (**f**) IBDQ-36 values at 0w and 12w of the same 17 UCc2 patients; p values were computed by pairwise paired or unpaired Wilcoxon tests (upper or lower values, respectively).

The effect of 12 weeks consumption of a fermented milk product (FMP) containing a consortium of 5 strains (Methods), which had shown anti-inflammatory effects in an experimental animal model^25^, was compared to that of an acidified milk product (MP) used as placebo. The hypothesis of this exploration study was that the FMP might improve microbial richness in UC patients on long-term remission. Thirty-one out of the UCc2 cohort participated, 17 and 14 being allocated to the FMP and MP arm, respectively. In parallel, a longitudinal comparison with 9 subjects from the healthy cohort was carried out (Extended Data Fig. 1a). Albeit randomization, the baseline characteristics of the two UCc2 groups differed in some respects (Supplementary Table 4) with somewhat higher calprotectin for those allocated to FMP than to MP arm (p = 0.065) and the extent of the disease (p = 0.016). Compliance of patients was satisfactory, as assessed by qPCRs fecal detection of FMP strains, used as proxy (Methods and Extended Data Fig. 8a–e). Richness did not change significantly in healthy controls (n = 9) during the 12-week follow-up (paired test, p = 0.29; Fig. 6a), but increased in UC patients on FMP (paired test, p = 0.008) or MP (paired test, p = 0.068). There was no effect on the evolution of the IBDQ-36 global index (Fig. 6b).

Moreover, considering patients from both arms (n = 31) at baseline, we observed higher richness in patients with IBDQ-36 score above the median compared to those below (Fig. 6c; p = 0.045). Concordantly (Fig. 6d), ETs3 individuals (n = 13) had higher IBDQ-36 score (p = 0.025) than the ETs1 individuals (n = 14). There was an association between microbiome and number of past relapses (Extended Data Fig. 9a,b): a trendy higher richness for patients who have relapsed twice of less (p = 0.072) and higher past relapses for those assigned to ETs1 compared to ETs3 individuals (p = 0.014). Terciles of WBC segregated well in a PCA analysis, based on 1133 MGS abundances (Extended Data Fig. 9c). Additionally (Extended Data Fig. 9d), patients above the median WBC value (> 6.35 × 10^9^/L; n = 15) increased their richness significantly whereas those below did not (paired tests, p = 0.0097 and p = 0.12 respectively). In terms of fecal calprotectin (Extended Data Fig. 9e), richness increase was observed for patients with values < 50 µg/g (n = 17) at baseline (paired test, p = 0.0015) while it was not significant for 50 to 250 or > 250 µg calprotectin level ( n = 6 and n = 7, respectively). Finally (Extended Data Fig. 9f), never-smokers (n = 12) compared to former-smokers (n = 14), increased their richness (paired tests, p = 0.0032 and p = 0.068 respectively) within the 12 weeks of monitoring, in contrast to active smokers (n = 5) who did not.

Because of its alleged role in UC^26^, we also focused on *Akkermansia muciniphila*. Besides the health *versus* UC distinction (Fig. 1f), in the pooled UC cohort (n = 65), the absence of this species was associated (p = 0.0066) with older age at diagnosis (A3 Montreal classification; Extended Data Fig. 10a) and tended to be linked with rate of past relapses > 0.5/year (p = 0.059; Extended Data Fig. 10b) while on the contrary, its detection was linked (p = 0.0017; Extended Data Fig. 10c) with extra-intestinal manifestations (EIMs, n = 13; details in Supplementary Table 1). In UCc2 (Extended Data Fig. 10d), absence of *A. muciniphila* at baseline was related to significantly lower richness but interestingly, richness in these negative patients (n = 23) increased (paired test, p = 0.0037) after the 12 weeks.

In patients with undetected *A. muciniphila* and fecal calprotectin < 250 µg/g at baseline (n = 17), while there was no longer any obvious imbalance at baseline (Supplementary Table 5), a significant increase of microbial richness was observed after the consumption in both FMP (n = 9) and MP (n = 8) groups (Fig. 6e) (paired tests, p = 0.02 and 0.0078 respectively), indicating that acidified milk also may have influenced richness. However, the richness gain was higher for the FMP group compared to MP group (difference of medians: 77 and 27 detected MGS respectively) and, importantly, IBDQ-36 score was significantly improved in the FMP group, while it was not in the MP group (paired tests, p = 0.042 and 0.2 respectively; Fig. 6f).

## Discussion

Our study demonstrates differences in gut microbiome between UC adult patients in remission and healthy controls. We found a significant loss of richness in patients and lower prevalence of the *Ruminococcus* species driven enterotype. Differences in gut microbial species abundance allow consistent discrimination between patients and healthy controls, with an AUC of 0.87 in ROC analysis, as also recently demonstrated by Zheng et al^8^. Loss of species was associated with the extent of the disease, being deeper in patients suffering from more extensive disease, and with relapse rate, being deeper in patients that relapsed more often. The conjunction of a high relapse rate and most extensive disease, defining an aggressive UC phenotype, was associated with the most marked richness loss. Depletion of the ETs3 was also associated with the aggressiveness of the disease.

Species richness was positively correlated with markers of subclinical inflammatory status of UC patients. This unanticipated observation was made in both UC cohorts, with different disease intensity profile, assessed cross-sectionally and longitudinally, via WBC or fecal calprotectin. This positive relationship, observed in the macro-inflammatory background of UC, is in sharp contrast with previous studies in metabolically unhealthy individuals, where an opposite relation was found, the high WBC counts being associated with low richness^15^. However, changes in the metabolic disorders develop slowly and steadily towards dysbiosis, whereas the gut microbiome in UC is unstable^27^ and exposed to temporal variability^7^ associated with the undulating course of the disease. In our follow-up study focusing on patients with high risk of disease flare, the individuals with baseline higher calprotectin levels had a significantly increased richness, at end time point, further supporting the observation that inflammation may be correlated with richness in at least some UC patients. Moreover, a higher richness, albeit not significant, appeared to be present in patients who progressed to overt clinical relapse, in comparison with their own baseline richness in remission or with those of patients who remained in persistent state of remission.

So, how to reconcile the highest loss of gut microbial richness observed in individuals in remission that relapsed most frequently in the past and that suffered from the most aggressive disease, with the increase of richness in individuals that have highest inflammation and will likely relapse? These apparently conflicting observations refer to different stages in the patients’ history. The former relates to past, of relapses and phenotype, and the latter to the present subclinical inflammatory level. We suggest that in UC, temporary increases of gut microbiome richness could trigger mucosal inflammation and thus lead to relapse. Subsequently, because of relapse, richness would be largely lost. Succession of past relapses likely leads to an imprint of lower richness.

In view of the high proportion of microbiome encoding macrolide degrading genes (Erm family) in patients with persistent state of remission, it is tempting to speculate that naturally occurring macrolides play a certain role in the onset of inflammation: this antibiotic family is being known for its anti-inflammatory properties. Additionally, tacrolimus, chemically a macrolide, which became part of UC therapeutics, especially in refractory patients^28^, could possibly select a beneficial microbiome encoding Erm family genes.

Finally, our study indicates that the trade-off between disease activity and gut microbial richness in UC patients is heterogeneous. In milder disease, characterized by rather low levels of inflammation, higher richness appears beneficial, as indicated by higher Inflammatory Bowel Diseases Quality of life score (IBDQ-36). Interestingly, consumption of a fermented milk product containing the anti-inflammatory strain *B. lactis* CNCM I-2494^29^ was associated with an increase in both richness and IBDQ-36 score in a subgroup of patients, having calprotectin below 250 µg/g and lacking *A. muciniphila*. In contrast, in patients with high calprotectin levels during clinical remission, richness increases may not be beneficial.

Possibly, our quantitative metagenomics approach to characterize the microbiome allowed, for a certain part, revealing these contrasting observations.

Clearly however, results of our work need to be repeated by further studies. These studies, at least both clinically (endoscopically as optimum) and microbiome-focused, involving larger numbers of more diverse UC patients followed longitudinally, should be encouraged to extend or challenge our findings.

## Methods

### Ethical aspects

The protocols associated with the study design were submitted and approved by the Ethics Committee of University Hospital Vall d’Hebron (CEIC-HUVH, Barcelona, Spain). Consequently, all methods involving human subjects were carried out in accordance with relevant guidelines and regulations, including institutional, national and international requirements. Ethical approval numbers are PR(AG)84/2007 and PR(AG)104/2009. The first refers to initial Ethics approval of all MetaHIT studies in Vall d’Hebron and the second is amendment. All volunteers received information concerning their participation in the study and informed consent was obtained from all subjects prior to their inclusion. For more information: fguarner@telefonica.net.

### Participants

Patients with ulcerative colitis (UC) attending the outpatient clinic of Hospital Vall d’Hebron were asked to give written consent to take part in this study. Eligible patients (inclusion criteria) were aged 18–75 years, had UC previously diagnosed by colonoscopy and histology, were in clinical remission as defined by a colitis activity index (CAI)^30^ of 3 or less, and had stable maintenance therapy either with mesalazine or azathioprine, or no treatment. Exclusion criteria included pregnancy or breast-feeding, severe concomitant disease involving the liver, heart, lungs of kidneys; treatment with steroids, cyclosporine, tacrolimus, anti-TNF drugs or topical anti-inflammatory preparations during the previous 3 months and treatment with antibiotics during the previous 4 weeks. Patients were recruited between April 2008 and April 2010; enrolment criteria for patients in the first cohort (UCc1) were clinical remission and stable medication for at least 3 months, but highly active disease in the past (> 3 relapses during the past 5 years) and, for the second cohort (UCc2), clinical remission as well as stable medication for at least one year with mild active UC in the previous 5 years (< 1 relapse/year).

Healthy controls (n = 56) were recruited among family relatives (n = 28) and unrelated healthy individuals (n = 28). Eligible subjects were aged 18–75 years and did not take any drug including antibiotics in the 4 months preceding sample and metadata collection.

### Metadata

#### Phenotypic and inflammation variables

All clinical characteristics were collected at the time of sampling.

For all individuals included in this study, demographic (age, gender) information and anthropometric measurements (weight, height and body mass index, BMI) as well as the usual smoking habit (current, former or never status) were recorded.

For each UC patient, clinical history of their disease activity about past relapses number as well as their illness duration were recorded to calculate their annual relapse rate. Also, for patients, UC disease specific variables were extracted from their clinical records: especially the age at diagnosis (Montreal classification^31^ as A1, A2, and A3, refers to age in years at the time of diagnosis below 16, between 17 and 40, and above 40, respectively) as well as for anatomic extent of the disease (E1, E2, and E3, denoting proctitis, left sided—distal colitis, and extensive – pancolitis, respectively). In addition, the presence of extra-intestinal manifestations (EIMs) of UC disease was also noted. Disease activity was assessed using the Colitis Activity index (CAI) scoring system (0: asymptomatic; 1–2: mildly symptomatic; 4–7: moderately symptomatic, and 8–13: highly symptomatic classes). Data collected in the anonymized patient files included blood cell counts and biochemistry.

As a maker of intestinal inflammation, fecal calprotectin was measured using a commercial enzyme-linked immunosorbent assay (ELISA) (Calprest; Eurospital SpA, Trieste, Italy), according to manufacturer’s instructions. Optical densities were read at 405 nm with a microplate ELISA reader (Multiskan EX; Thermo Electron Corporation, Helsinki, Finland). Samples were tested in duplicate and results were calculated from a standard curve and expressed as µg/g stool.

#### Genotyping

Individuals in UC cohort 1 (n = 31) and their healthy relatives (n = 28) were genotyped for SNPs relevant to UC and common among Spanish patients. Assays were performed using the TaqMan genotyping protocols by Applied Biosystems (Foster City, California, US). Briefly, the assays use TaqMan 5´‑nuclease chemistry to amplify and detect specific polymorphisms in purified genomic DNA samples. A single nucleotide polymorphism (SNP) is addressed in each assay and consists of two sequence-specific primers and two TaqMan minor groove binder probes with non-fluorescent quenchers. One probe is labeled with VIC dye to detect the Allele 1 sequence; the second probe is labeled with FAM dye to detect the Allele 2 sequence. Purified DNA extracted from individuals’ blood samples were assayed for RNF 186 (rs6426833), CARD9 (rs4077515), LOC105369818 (rs971545), IL23R (rs2201841), LOC107984526 or IL26 (rs1558744), and PUS10 (rs13003464) genes.

#### Randomized placebo control probiotic milk products intake exploratory study

The study was single center, randomized, double-blind, controlled with a parallel group design^32,33^. This study had no clinical endpoint but aimed at evaluating the effect of consumption of a fermented milk product (FMP) on both gut microbiota richness and UC patient quality of life, as assessed using a validated questionnaire (explained below). From the thirty-four patients enrolled in UCc2, thirty-one volunteered (three patients had declined) to participate in the trial (UCc2 trial) and were randomized (random drawing of a sealed envelope) to receive FMP (n = 17) or the control product (n = 14), an unfermented acidified milk vehicle (MP). Additional nine healthy subjects were included in a parallel arm as controls for temporal variability of microbiome composition (Extended Data Fig. 1a). All participants were asked to avoid fermented dairy products during the 12-week study period. After a one-week wash-out period (verified declaratively), once their samples (week 0 or baseline) had been taken, 31 patients (per protocol) consumed (125 g/serving) twice a day either the FMP or the MP for 12 weeks. FMP contained 1.25 × 10^10^ colony forming units per serving (cfu/serving) Bifidobacterium animalis subs. lactis strain CNCM I-2494, 1.2 × 10^9^ cfu/serving of Streptococcus thermophilus CNCM I-1630 Lactobacillus delbrueckii subs. bulgaricus CNCM I-1632 and CNCM I-1519 and Lactococcus lactis CNCM I-1631. The MP was a nonfermented acidified milk product, thus without probiotics, and with low lactose content (< 4gr/serving). Each serving contained either 125 g of FMP or MP and were provided by Danone Research (Palaiseau, France); packaged in boxes, the portions (either FMP or MP) were sent to included patients’ home, where they were stored in the refrigerator until consumed.

Baseline and follow-up medical visits were scheduled: in addition to collecting samples, they were intended to ensure that patients were adhering to the dairy product intake, to assess their clinical condition and, particularly, to evaluate their quality of life.

For this FMP versus MP trial, the patients’ quality of life was therefore assessed using the specific 36 items Inflammatory Bowel Diseases Quality (IBDQ-36) questionnaire, which was completed at weeks 0 and 12. Briefly, this is a self-administered questionnaire that had previously been translated and validated for its use in Spanish^34,35^. It includes items grouped into five domains of health: digestive (or bowel) symptoms, systemic symptoms, emotional symptoms (or function), functional activity (in other words, impairment) and social involvement (or impairment). Responses were scored on a 7-point Likert scale, in which 7 corresponds to the highest level of functioning. Finally, the IBDQ-36 global index was defined as the mean of the 5 aforementioned responses.

#### Quantitative polymerase chain reactions analysis of fecal levels of FMP strains

Determination of bacterial concentration is based on the quantification of DNA molecules using either primers targeting 16S rRNA sequences or CRISPR regions for strains specific detections as previously described on Bifidobacterium animalis subs. lactis strain CNCM (Collection Nationale de Cultures de Microorganismes) I-2494, Streptococcus thermophilus CNCM I-1630, Lactobacillus delbrueckii subs. bulgaricus CNCM I-1632 and CNCM I-1519 and lastly Lactococcus lactis CNCM I-1631^36^.

#### Clinical metadata mining

Our strategy to mine the Metadata originated with clinical numeric variables discretization in two different ways: either we considered it was possible to use some cutoff values (cutoff) from medical literature as a cleaver (< or > to this cutoff), or we employed by default the median directly from the numeric variable (as ≤ or > to this median) for each cohort or subgroup. Thus, for fecal calprotectin, although we acknowledge no preexisting formal validation, we decided to opt for 50 µg/g and 250 µg/g or 100 µg/g as cutoff values as these thresholds were commonly applied (Lin et al^22^ and Mosli et al^37^). Similarly, we chose 25 kg/m^2^ of body mass index (BMI) cutoff value according to the work from Stabroth-Akil et al^38^.

### Metagenomic data production

According to the recommendations of the International Human Microbiome Standards (IHMS; https://human-microbiome.org/), fecal samples collected were immediately frozen by the participants in their home freezer at − 20 °C and later brought to the laboratory in a freezer pack, where they were stored at − 80 °C following IHMS SOP 04 V2. DNA extraction from fecal samples was performed following IHMS SOP 07 V2. The evaluation of this protocol is available elsewhere^39^. The DNA concentration and its molecular size were estimated by nanodrop (Thermo Scientific) and agarose gel electrophoresis respectively. Shotgun sequencing and its library construction was carried out on Illumina platform. Descriptive statistics of the raw sequences produced are published elsewhere^12,13,40^. All sequenced fecal DNA samples of this study (n = 186) have made up the material to construct as reference the integrated gene catalog for human gut microbiome as published^12^ in 2014 by the MetaHIT consortium. In brief, this catalog comprised 9,879,896 (9.9 M) reference genes and was built from the metagenomic sequencing of the faeces of 1,018 individuals of various geographic origin (Europe, USA, China), healthy or not (suffering mainly from obesity, diabetes, Crohn’s disease or UC). Through MOCAT^41^ processing pipeline, the majority of these genes were predicted on contigs from de novo assembly of metagenomic samples associated with microbiome study projects (mostly MetaHIT and HMP^42^). The other genes came from 511 genomes of gut-related prokaryotic species. This catalog was non redundant due to filtering at 95% nucleic acid identity and 90% coverage using CD-HIT^43^ clustering.

### Metagenomic gene abundance profiling

A metagenomic gene abundance table was generated using MOCAT^44^. Raw sequencing reads were trimmed (option read_trim_filter) using a quality and length cut-off of 20 and 45 bp, respectively. Trimmed reads were subsequently screened against a database of Illumina adapters (option screen_fastafile) and the human genome sequence (version hg19) using a 90% identity cut-off (option screen). Gene count profiles were generated by aligning (option screen) the quality-controlled sequencing reads from each metagenome to the reference gene catalogue^12^ using an alignment length and identity cut-off of 45% and 95%, respectively. Gene counts were calculated as a function of uniquely mapped reads and distributed contributions of non-uniquely mapped reads as described in^45^.

### Structuration into MetaGenomic Species

To map out the metagenomic species (MGS), co-abundant consistent genebags in short, MSPminer^14^ software, was used. By binning co-abundant genes across metagenomic samples, its algorithm relies on a robust measure of proportionality coupled with an empirical classifier to group and distinguish not only species core genes but accessory genes also. Applied to the metagenomic gene count table of the 1267 shotgun sequenced stool samples after mapping onto the 9.9 M catalog, 3,288,928 genes were clustered into “msp_” or metagenomic species pan-genomes, in other words, MGS but refined in comparison with their initial delineation from CAGs^13^ (co-abundance gene groups). Briefly, in output of MSPminer, each MGS is a list of genes subdivided into three main parts: core, accessory or shared. Core genes are co-abundant and co-present in all metagenomic samples where a given MGS is detected whereas accessory genes are also co-abundant but co-present only in a subset of these samples. Shared genes are not specific of this MGS.

### Taxonomic assignation of MGS

As previously described^14^, MGS taxonomic annotation was performed by aligning all core and accessory genes against nt and WGS (version of October 2018 restricted to the taxa Bacteria, Archaea, Fungi, Viruses and Blastocystis) using blastn^46^ (version 2.7.1, task = megablast, word_size = 16). The best 30 hits for each gene were kept. A species-level assignment was given if > 50% of the genes matched the RefSeq reference genome of a given species, with a mean identity ≥ 95% and mean gene coverage ≥ 90%. The remaining MGS were assigned to a higher taxonomic level (genus to superkingdom), if more than 50% of their genes had the same annotation. Together with this taxonomic assignation process, for each MGS, co-abundance signal was manually checked and was eventually confronted with previously generated consistent gene clusters^13^if available. Moreover, some MGS still had a "CAG:" assignation. It meant that they had also been consistently assembled as metagenomic associated (draft) genomes, and deposited in the NCBI database, often with no annotation at the time of their submission. Phylogroups were determined after construction of a phylogenetic tree as described elsewhere^14^. Typically, phylogroup 1 contained the type strain whose MGS had taken the name. This exploration of phylogeny was also an integral part of the various manual verifications. In the end, it resulted in an “improved” dataset of 1,776 MGS which we mined for our analysis.

### Metagenomic gene count table post-processing

The gene abundance table was post-processed for downsizing and then normalization using the MetaOMineR (momr) R package^47^. Taking into account sequencing depth and technical variability, samples were downsized to 22 million (22 M) mapped reads as described previously^15^. The abundance of each gene was then normalized by dividing the number of reads that are mapped to a given gene by its nucleotide length and by the total number of reads from a given sample. The resulting rarefied and normalized abundance table was finally reported in frequency. It was this post-processed metagenomic gene count table we next used to estimate the abundance of MGS. For that, the relative abundance of MGS was determined as mean count of the 90% of the first (basically core) 50 genes of each MGS. In other words, an MGS was considered detected in a given sample if at least 10% of these 50 genes had a non-zero value. This is how we built the MGS abundance table, or its normalized derivative (where the sum of all MGS in a given sample is equal to 1).

### Richness and enterotyping

Species richness, or MGS count variable, was determined by summing among the 1,776 MGS detected in each metagenomic sample. For enterotyping, the genus abundance table, which was constructed by summing in each sample all MGS annotated with a same genus, was used in input for DMM approach^17^ by means of the DirichletMultinomial R package^48^. The same method was applied to determine enterotypes (ETs) except that it was performed directly from MGS counting table. ETs drivers taxa identification as well as categorical sample tagging was carried out as described elsewhere^16–18^.

### Functional annotation

The integrated gene catalog was annotated for the Antibiotic Resistant Determinants (ARD) described in Mustard database (v1.0)^24,49^. Protein sequences were aligned against 9 462 ARD sequences using blastp 2.7.1 + (option -evalue 1e−5). Best-hit alignments were filtered for identity >  = 95% and bidirectional alignment coverage >  = 90% (at query and subject level), giving a list of 7,251 ARD candidates belonging to 30 families. Annotation of the carbohydrate-active enzymes (CAZymes) was performed by comparing the predicted protein sequences to those in the CAZy database and to Hidden Markov models (HMMs) built from each CAZy family^50^, following a procedure previously described for other metagenomics analyses^51^.

Activity and even expression of ARD or CAZyme candidates being unknown, these functions were evaluated as a fraction of microbiome: for each sample, the detected species were screened at the strain level for the presence of the tested function, and the function abundance was evaluated summing the signal of all positive species.

### Data and metadata analysis

As all the questions formulated in this study had been placed in a purely exploratory objective by the MetaHIT consortium, the exclusive purpose of our analysis was descriptive. The significance thresholds used were ≤ 0.05 for p values and ≤ 0.1 (q values) for Benjamini-Hochberg^52^ false discovery rate (FDR) q values. Apart from most Supplementary Tables, multiple correction was applied whenever more than 10 statistical tests were performed simultaneously. Alongside our graphical representations, we labelled each of our results with their p values only, unless exception. All statistical tests were two-sided. If missing values (MV) were present (especially for clinical metadata), statistical tests were performed by omitting them. All analyses were performed in R^53^ (version 3.6.0, Vienna, Austria). As and when they were used, we mentioned the R packages that we employed as well as some of their respective functions (in italic) otherwise those with occasional use for one or the other figure were: shape^54^, diagram^55^, reshape2^56^, ggplot2^57^, cowplot^57^, ggdendro^58^ and ggplotify^59^. Supplementary Tables were generated thanks to flextable^60^ and officer^61^ R packages.

#### Bivariate exploration

Wilcoxon rank sum test (Wilcoxon signed rank test, with paired = TRUE argument, as appropriate in longitudinal data), or Kruskal–Wallis (KW) rank sum test where appropriate, was carried out when confronting a numerical variable with another categorical represented graphically represented by boxplot (base *boxplot* function with notch = TRUE argument). The boxes were the interquartile range (IQR), from the first and third quartiles, and the inside line was the median. The whiskers denoted the lowest and highest values within 1.5 IQR from the first and third quartiles. The black points (joined by gray cords for each pair longitudinally followed) represented each individual value with outliers beyond the whiskers. The notches showed the 95% confidence (CI) for the medians. Cliff’s Delta (CliffsD) effect size was computed with *cliff.delta* function (default settings) from the effsize R package^62^. Its magnitude was assessed using the thresholds provided in^63^, |CliffsD|< 0.147 “negligible”, |CliffsD|< 0.33 “small”, |CliffsD|< 0.474 “medium”, otherwise “large”. To study the independence of two categorical variables, a barplot was displayed. In this case, Fisher’s exact test was used. An odd ratio with its confidence interval was also calculated if it was applied on a 2 × 2 contingency table. Finally, if the comparison concerned two numeric variables, Spearman’s rho correlation coefficient was calculated as well as its probability to be different from zero. The respective values of the two vectors were then displayed as scatterplot and the linear regression line was systematically drawn for information purposes.

#### Multivariate factorial analysis

While the vast majority of our descriptive report relied on a bivariate statistical approach that used the normalized MGS abundance table, the multivariate ordination analysis (classification modelling included) was performed with the MGS abundance table but signal weighted and log10 transformed (and also beforehand filtered if deemed necessary). For this table, each abundance vector was multiplied by the product of median abundance and occurrence in metagenomic samples of interest where a given MGS was detected, defining the signal of this MGS to be weighted. In addition to the theoretical interest of giving even more weight to detected species that are more abundant and also more occurrent or more present, the benefit of this weighting step according to the signal of each MGS was emphasized during the modelling process (detailed below). In practice, starting from the MGS abundance matrix, base 10 logarithm (as variance stabilization) function was applied to each of its values added with a pseudocount (defined as the non-zero minimum), and this, after that each abundance vector had been multiplied by its corresponding MGS signal parameter. This signal weighted and log10 transformed counting matrix was named weighted MGS abundance table. To represent ET(s), Bray Curtis dissimilarity was generated using vegdist function in the vegan R package^64^. Thereafter, the distances calculated from our weighted (detected) MGS abundance table were expressed by principal coordinate analysis (PCoA). Finally, in order to reveal at best the inter-group total variance or inertia, between-class analysis (bca) was then applied with its associated p value resulting from a Monte-Carlo permutation test (randtest function with nrepet = 999 and alter = "two-sided" arguments) on this between-class inertia (in percentages). All functions used for multi-dimensional exploration, with their statistical or graphical outputs, were mostly implementations of the ade4 R package^65^ or from FactoMineR^66^. Also, in the framework of Euclidian exploratory methods, once the variables have been preprocessed by centering and scaling, principal component analysis (PCA) was considered most often. To present multivariate analysis results, the first factorial plan, by definition the one with the highest amount of information (expressed as percentage of inertia for each axis), was exhibited with its first (horizontal) and second (vertical) axis drawn in dotted lines. Drawing a graph of individuals, each of plotted point, with their coordinates, was also colored according its categorical belonging (instrumental variable for between-class analysis). While the dots seemed to outline a singular shape for each colored cloud, all individual point were joined to the label of their respective belonging, which was, for each category, positioned at barycenter, and circumscribed by an ellipse. The ellipses, whose length of their axes was 2.5 times the standard deviation of the coordinates on this first plan, and thus contained 95% of the points of each cloud, were to be considered here as a simple graphical summary (and therefore in no case as ellipses of confidence).

#### Classification modelling

Consistently in a descriptive context, we built a classifier based on weighted MGS abundance table to discriminate healthy subjects (n = 56) from UC patients (n = 65). In order to justify the choice of classification algorithm, we proceeded to an empirical comparison of classifiers. For this, we employed R packages designed for the construction of models from both statistical or machine learning approaches, all interfaced with the caret R package^67^. We performed our modeling process according to the principles and methods (details about classifiers included) that have been explained elsewhere^68–70^. As performance metric, we adopted AUC (ROC), by using ci.auc function (default setting) from pROC R package^71^. In addition to performance, we also regarded the considered features and their respective importance using varImp function (default setting) from caret, variable importance which were calculated by different classifiers. As a preliminary step, we employed nearZeroVar function (default setting), from caret package also, to filter the number of variables or features to consider. This resulted in weighted MGS abundance table with 684 features out of the 1484 MGS detected among the 121 individuals of the cohort. In the first stage we preselected classifier modeling methods, based on their ease of use (proper grid with boundaries of hyperparameters to tune, for example), particularly with the train function (again from caret package). Three were from the field of statistics and two from machine learning. The former included partial least square discriminant analysis (pls-DA), elastic net or regularized form of logistic regression (glmnet), and random forests (rf), the latter included support vector machine (svm) with radial basis function kernel and neural networks (nnet). Consistent with the learning strategy, the data have been divided into train (75%, n = 91) and test (25%, n = 30) sets and all classifiers were run in a 100-repeated process. On training dataset, a 10-Fold cross-validation (CV) was run for hyperparameters tuning, using tenfold train data split into 10 sets of roughly the same size. The model was fitted on 90% and the remaining 10% was used to estimate accuracy. This process continued round-robin 9 more times. Based on the selected hyperparameters, a model was fitted on the whole training set and applied on the test set. Iteratively, for each classifier, varImp was performed to the output of the train function. For each pair of training and test (or out-of-sample) sets, the performance was quantified (ci.auc function). Randomization seeds were stable across classifiers within an iteration (100 repetitions = 100 data points per pair for each of the five classifiers) to allow comparison. Regarding the results, both on train and test datasets (Extended Data Fig. 3a,b), the performance of each classifier was not highly different. Specifically, on training data, pls-DA yielded the highest median AUC value (0.88) followed closely by svm (0.86), nnet and glmnet (0.85), discriminant power of rf (0.80) being clearly lower. Similar results were obtained on test data—for instance, the median AUC values were the same for pls-DA (0.88), and somewhat improved for svm (0.88). Concerning the importance of variables (varImp function output), expressed as numeric values scaled between 0 and 100, we noticed a very close distance between the pls-DA and nnet (Extended Data Fig. 3c) in a principal component analysis based on the table of the medians of varImp (from 100 iterations) for the 684 features used to fit train models. Consistently, for pls-DA and nnet the most important features had similar order and value for their varImp (medians on 100 repetitions, Extended Data Fig. 3e). Convergence of features selected by a statistical learning method and a machine learning tool is reassuring, as it indicates that robust species-level microbiome differences exist between healthy individuals and UC patients and can be used for stratification. The same workflow was applied also to MGS abundance table (log10 transformed but without signal weighting). In terms of performance, the same order of AUC values and the same ranking was observed for different classifiers but somewhat inferior: on training data, median AUC values were 0.87 for pls-DA, 0.84 with nnet or svm and glmnet, 0.80 in rf while on test data, performances were 0.87 with pls-DA and svm, 0.85 for nnet, 0.81 pour rf et glmnet. Furthermore, pls-DA and nnet were less close in a PCA analysis (Extended Data Fig. 3d). The improvement with the weighted MGS abundance led us to use this signal in multivariate factorial analyses. In conclusion, comparison of modeling algorithms led us to give preference to partial least square discriminant analysis, as, in addition to best performance, it had the advantage of a better interpretability^69^ compared to an artificial intelligence approach such as neural networks. 1,000 iterations were carried out and the result is displayed in Fig. 1e,f.

## Supplementary Information

Supplementary Information.

Supplementary Material 2

## Data Availability

During peer review process, any supplementary information can be added at the request of the evaluators. In the same way, data availability as well as the R code used will be made accessible with the publication. Metagenomic study accession (Project IDs) are [PRJEB1220] (https:/www.ebi.ac.uk/ena/browser/view/PRJEB1220) and [PRJEB5224] (https:/www.ebi.ac.uk/ena/browser/view/PRJEB5224) (Details in file “metaHIT_UC_accession_number_informations.xlsx”.)

## References

[1] Bernstein, C. N. & Shanahan, F. Disorders of a modern lifestyle-reconciling the epidemiology of inflammatory bowel diseases. *Gut* **57**, 1185–1192 (2008). 18515412 10.1136/gut.2007.122143

[2] Shouval, D. S. & Rufo, P. A. The role of environmental factors in the pathogenesis of inflammatory bowel diseases: A review. *JAMA Pediatr.* **171**, 999–1005 (2017). 28846760 10.1001/jamapediatrics.2017.2571

[3] Schirmer, M. et al. Compositional and temporal changes in the gut microbiome of pediatric ulcerative colitis patients are linked to disease course. *Cell Host Microbe* **24**, 600-610.e4 (2018). 30308161 10.1016/j.chom.2018.09.009 PMC6277984

[4] Jostins, L. et al. Host-microbe interactions have shaped the genetic architecture of inflammatory bowel disease. *Nature* **491**, 119–124 (2012). 23128233 10.1038/nature11582 PMC3491803

[5] Sartor, R. B. & Wu, G. D. Roles for intestinal bacteria, viruses, and fungi in pathogenesis of inflammatory bowel diseases and therapeutic approaches. *Gastroenterology* **152**, 327-339.e4 (2017). 27769810 10.1053/j.gastro.2016.10.012 PMC5511756

[6] Franzosa, E. A. et al. Gut microbiome structure and metabolic activity in inflammatory bowel disease. *Nat. Microbiol.* **4**, 293–305 (2019). 30531976 10.1038/s41564-018-0306-4 PMC6342642

[7] Lloyd-Price, J. et al. Multi-omics of the gut microbial ecosystem in inflammatory bowel diseases. *Nature* **569**, 655–662 (2019). 31142855 10.1038/s41586-019-1237-9 PMC6650278

[8] Zheng, J. et al. Noninvasive, microbiome-based diagnosis of inflammatory bowel disease. *Nat. Med.*10.1038/s41591-024-03280-4 (2024). 39367251 10.1038/s41591-024-03280-4 PMC11645270

[9] Gevers, D. et al. The treatment-naive microbiome in new-onset Crohn’s disease. *Cell Host Microbe* **15**, 382–392 (2014). 24629344 10.1016/j.chom.2014.02.005 PMC4059512

[10] Lepage, P. et al. Twin study indicates loss of interaction between microbiota and mucosa of patients with ulcerative colitis. *Gastroenterology* **141**, 227–236 (2011). 21621540 10.1053/j.gastro.2011.04.011

[11] Qin, N. et al. Alterations of the human gut microbiome in liver cirrhosis. *Nature* **513**, 59–64 (2014). 25079328 10.1038/nature13568

[12] Li, J. et al. An integrated catalog of reference genes in the human gut microbiome. *Nat. Biotechnol.* **32**, 834–841 (2014). 24997786 10.1038/nbt.2942

[13] Nielsen, H. B. et al. Identification and assembly of genomes and genetic elements in complex metagenomic samples without using reference genomes. *Nat. Biotechnol.* **32**, 822–828 (2014). 24997787 10.1038/nbt.2939

[14] Plaza Oñate, F. et al. MSPminer: abundance-based reconstitution of microbial pan-genomes from shotgun metagenomic data. *Bioinformatics*10.1093/bioinformatics/bty830 (2018). 10.1093/bioinformatics/bty830 PMC6499236 30252023

[15] Le Chatelier, E. et al. Richness of human gut microbiome correlates with metabolic markers. *Nature* **500**, 541–546 (2013). 23985870 10.1038/nature12506

[16] Arumugam, M. et al. Enterotypes of the human gut microbiome. *Nature* **473**, 174–180 (2011). 21508958 10.1038/nature09944 PMC3728647

[17] Holmes, I., Harris, K. & Quince, C. Dirichlet multinomial mixtures: Generative models for microbial metagenomics. *PLoS ONE*10.1371/journal.pone.0030126 (2012). 22319561 10.1371/journal.pone.0030126 PMC3272020

[18] Costea, P. I. et al. Enterotypes in the landscape of gut microbial community composition. *Nat. Microbiol.* **3**, 8–16 (2017). 29255284 10.1038/s41564-017-0072-8 PMC5832044

[19] Magro, F. et al. Third European Evidence-based Consensus on Diagnosis and Management of Ulcerative Colitis. Part 1: Definitions, Diagnosis, Extra-intestinal Manifestations, Pregnancy, Cancer Surveillance, Surgery, and Ileo-anal Pouch Disorders. *J Crohns Colitis* **11**, 649–670 (2017). 28158501 10.1093/ecco-jcc/jjx008

[20] Da Silva, B. C., Lyra, A. C., Rocha, R. & Santana, G. O. Epidemiology, demographic characteristics and prognostic predictors of ulcerative colitis. *World J. Gastroenterol.* **20**, 9458–9467 (2014). 25071340 10.3748/wjg.v20.i28.9458 PMC4110577

[21] McGovern, D. P. B. et al. Genome-wide association identifies multiple ulcerative colitis susceptibility loci. *Nat. Genet.* **42**, 332–337 (2010). 20228799 10.1038/ng.549 PMC3087600

[22] Lin, J. F. et al. Meta-analysis: Fecal calprotectin for assessment of inflammatory bowel disease activity. *Inflamm. Bowel Dis.* **20**, 1407–1415 (2014). 24983982 10.1097/MIB.0000000000000057

[23] Wright, E. K. Calprotectin or lactoferrin: Do they help. *Dig. Dis.* **34**, 98–104 (2016). 26982329 10.1159/000442935

[24] Ruppé, E. et al. Prediction of the intestinal resistome by a three-dimensional structure-based method. *Nat. Microbiol.* **4**, 112–123 (2019). 30478291 10.1038/s41564-018-0292-6

[25] Veiga, P. et al. Bifidobacterium animalis subsp lactis fermented milk product reduces inflammation by altering a niche for colitogenic microbes. *Proc. Natl. Acad. Sci. U S A* **107**, 18132–18137 (2010). 20921388 10.1073/pnas.1011737107 PMC2964251

[26] Earley, H. et al. The abundance of Akkermansia muciniphila and its relationship with sulphated colonic mucins in health and ulcerative colitis. *Sci. Rep.*10.1038/s41598-019-51878-3 (2019). 31666581 10.1038/s41598-019-51878-3 PMC6821857

[27] Martinez, C. et al. Unstable composition of the fecal microbiota in ulcerative colitis during clinical remission. *Am. J. Gastroenterol.* **103**, 643–648 (2008). 18341488 10.1111/j.1572-0241.2007.01592.x

[28] Liu, Y. J. et al. Pooled analysis of the comparative efficacy between tacrolimus and infliximab for ulcerative colitis. *Medicine*10.1097/MD.0000000000011440 (2018). 30095612 10.1097/MD.0000000000011440 PMC6133612

[29] Martín, R. et al. Bifidobacterium animalis ssp lactis CNCM-I2494 restores gut barrier permeability in chronically low-grade inflamed mice. *Front. Microbiol.* **7**, 608 (2016). 27199937 10.3389/fmicb.2016.00608 PMC4858658

[30] Walmsley, R. S., Ayres, R. C. S., Pounder, R. E. & Allan, R. N. A simple clinical colitis activity index. *Gut* **43**, 29–32 (1998). 9771402 10.1136/gut.43.1.29 PMC1727189

[31] Satsangi, J., Silverberg, M. S., Vermeire, S. & Colombel, J. F. The Montreal classification of inflammatory bowel disease: Controversies, consensus, and implications. *Gut* **55**, 749–753 (2006). 16698746 10.1136/gut.2005.082909 PMC1856208

[32] Veiga, P. et al. Changes of the human gut microbiome induced by a fermented milk product. *Sci. Rep.* **4**, 1–9 (2014). 10.1038/srep06328 PMC4160712 25209713

[33] Agrawal, A. et al. Clinical trial: The effects of a fermented milk product containing *Bifidobacterium lactis* DN-173 010 on abdominal distension and gastrointestinal transit in irritable bowel syndrome with constipation. *Aliment. Pharmacol. Ther.* **29**, 104–114 (2008). 18801055 10.1111/j.1365-2036.2008.03853.x

[34] Casellas, F., Vilaseca, J., Malagelada, J.-R., López-Vivancos, J. & Badia, X. Validation of the Spanish version of the Inflammatory Bowel Disease Questionnaire on ulcerative colitis and Crohn’s disease. *Digestion* **60**, 274–280 (1999). 10343142 10.1159/000007670

[35] Casellas, F., Alcalá, M. J., Prieto, L., Miró, J. R. A. & Malagelada, J. R. Assessment of the influence of disease activity on the quality of life of patients with inflammatory bowel disease using a short questionnaire. *Am. J. Gastroenterol.* **99**, 457–461 (2004). 15056085 10.1111/j.1572-0241.2004.04071.x

[36] McNulty, N. P. et al. The impact of a consortium of fermented milk strains on the gut microbiome of gnotobiotic mice and monozygotic twins. *Sci. Transl. Med.* **3**, 106 (2011). 10.1126/scitranslmed.3002701 PMC3303609 22030749

[37] Mosli, M. H. et al. C-reactive protein, fecal calprotectin, and stool lactoferrin for detection of endoscopic activity in symptomatic inflammatory bowel disease patients: A systematic review and meta-analysis. *Am. J. Gastroenterol.* **110**, 802–819 (2015). 25964225 10.1038/ajg.2015.120

[38] Stabroth-Akil, D., Leifeld, L., Pfützer, R., Morgenstern, J. & Kruis, W. The effect of body weight on the severity and clinical course of ulcerative colitis. *Int. J. Colorectal Dis.* **30**, 237–242 (2015). 25392256 10.1007/s00384-014-2051-3

[39] Costea, P. I. et al. Towards standards for human fecal sample processing in metagenomic studies. *Nat. Biotechnol.* **35**, 1069–1076 (2017). 28967887 10.1038/nbt.3960

[40] Qin, J. et al. A human gut microbial gene catalogue established by metagenomic sequencing. *Nature* **464**, 59–65 (2010). 20203603 10.1038/nature08821 PMC3779803

[41] Kultima, J. R. et al. MOCAT: A Metagenomics Assembly and Gene Prediction Toolkit. *PLoS ONE* **7**, 1–6 (2012). 10.1371/journal.pone.0047656 PMC3474746 23082188

[42] The Human Microbiome Project consortium. Structure, function and diversity of the healthy human microbiome. *Nature* **486**, 207–214 (2012). 22699609 10.1038/nature11234 PMC3564958

[43] Fu, L., Niu, B., Zhu, Z., Wu, S. & Li, W. CD-HIT: Accelerated for clustering the next-generation sequencing data. *Bioinformatics* **28**, 3150–3152 (2012). 23060610 10.1093/bioinformatics/bts565 PMC3516142

[44] Kultima, J. R. et al. MOCAT2: A metagenomic assembly, annotation and profiling framework. *Bioinformatics* **32**, 2520–2523 (2016). 27153620 10.1093/bioinformatics/btw183 PMC4978931

[45] Forslund, K. et al. Disentangling type 2 diabetes and metformin treatment signatures in the human gut microbiota. *Nature* **528**, 262–266 (2015). 26633628 10.1038/nature15766 PMC4681099

[46] Altschul, S. F., Gish, W., Miller, W., Myers, E. W. & Lipman, D. J. Basic local alignment search tool. *J. Mol. Biol.* **215**, 403–410 (1990). 2231712 10.1016/S0022-2836(05)80360-2

[47] Prifti, E. & Le Chatelier, E. momr: Mining Metaomics Data (MetaOMineR). R package version 1.1 (2015).

[48] Martin, M. DirichletMultinomial: Dirichlet-Multinomial Mixture Model Machine Learning for Microbiome Data. R package version 1.24.1 (2019).

[49] EvoTAR consortium. Antimicrobial Resistance Determinants (ARDs). http://www.mgps.eu/Mustard.

[50] Lombard, V., Golaconda Ramulu, H., Drula, E., Coutinho, P. M. & Henrissat, B. The carbohydrate-active enzymes database (CAZy) in 2013. *Nucleic Acids Res.* **42**, 490–495 (2014). 10.1093/nar/gkt1178 PMC3965031 24270786

[51] Svartström, O. et al. Ninety-nine de novo assembled genomes from the moose (*Alces alces*) rumen microbiome provide new insights into microbial plant biomass degradation. *ISME J.* **11**, 2538–2551 (2017). 28731473 10.1038/ismej.2017.108 PMC5648042

[52] Benjamini, Y. & Hochberg, Y. Controlling the false discovery rate: A Practical and powerful approach to multiple testing. *J. R. Stat. Soc. B* 289–300 (1995).

[53] R Core Team. R: A Language and Environment for Statistical Computing. https://www.r-project.org/ (2019).

[54] Soetaert, K. shape: Functions for Plotting Graphical Shapes, Colors. R Package 1.4.4 (2018).

[55] Soetaert, K. *diagram: Functions for Visualizing Simple Graphs (Networks), Plotting Flow Diagrams. R Package 1.6.4* (2017).

[56] Wickham, H. Reshaping Data with the reshape R Package. *J. Stat. Soft.* **21**, 1–20 (2007).

[57] Wickham, H. *Ggplot2: Elegant Graphics for Data Analysis* (Springer-Verlag, 2016).

[58] de Vries, A. & Ripley, B. D. ggdendro: Create Dendrograms and Tree Diagrams Using ggplot2. R Package 0.1-20 (2016).

[59] Yu, G. ggplotify: Convert Plot to ‘grob’ or ‘ggplot’ Object. R Package 0.0.3 (2018).

[60] Gohel, D. flextable: Functions for Tabular Reporting. R Package 0.5.5 (2019).

[61] Gohel, D. officer: Manipulation of Microsoft Word and PowerPoint Documents. R Package 0.3.5 (2019).

[62] Torchiano Marco. effsize: Efficient Effect Size Computation. R package 0.7.6. (2018).

[63] Romano, J., Kromrey, J. D., Coraggio, J., Skowronek, J. & Devine, L. Exploring methods for evaluating group differences on the NSSE and other surveys: Are the t-test and Cohen’s d indices the most appropriate choices? *Annual meeting of the Southern Association for Institutional Research* 14–17 (2006).

[64] Oksanen, J. *et al.* vegan: Community Ecology. R Package 2.5-6 (2019).

[65] Dufour, A.-B. & Stéphane, D. The ade4 Package: Implementing the Duality Diagram for Ecologists. *J. Stat. Softw.* **22**, 1–20 (2007).

[66] Lê, S., Josse, J. & Husson, F. FactoMineR: A package for multivariate analysis. *J. Stat. Softw.* **25**, 1–18 (2008).

[67] Kuhn, M. *et al.* caret: Classification and Regression Training. R Package 6.0-84 (2019).

[68] Kuhn, M. & Johnson, K. *Applied Predictive Modeling* (Springer Science+Business Media, 2013).

[69] James, G., Witten, D., Hastie, T. & Tibshirani, R. *An Introduction to Statistical Learning: With Application in R* (Springer-Verlag, 2013).

[70] Deist, T. M. et al. Machine learning algorithms for outcome prediction in (chemo)radiotherapy: An empirical comparison of classifiers. *Med. Phys.* **45**, 3449–3459 (2018). 29763967 10.1002/mp.12967 PMC6095141

[71] Robin, X. et al. pROC: an open-source package for R and S+ to analyze and compare ROC curves. *BMC Bioinform.* **12**, 77 (2011). 10.1186/1471-2105-12-77 PMC3068975 21414208

