# Supplementary material for: Fecal microbiome of patients with ulcerative colitis reflects their phenotype and inflammatory level
Source: Sci Rep. 2026 May 19;16:25249. doi: 10.1038/s41598-026-44895-6 (PMC13469089; doi:10.1038/s41598-026-44895-6)
Supplement: Supplementary file 1 [file 41598_2026_44895_MOESM1_ESM.docx]

**Supplementary information**

**Extended data figures**

**Extended Data Fig. 1: MetaHIT IBD ulcerative colitis (UC) study diagram.**

Three cross-sectional cohorts (spheres) were constituted, two of patients differing especially by the importance of past relapses, higher in UC cohort 1 (UCc1) than in UC cohort 2 (UCc2), and one of healthy subjects (H). Individuals were followed longitudinally: UCc1 time course (TC) patients until relapse or for a year if remaining in remission; Ucc2 trial patients were randomly assigned to two groups, one consuming a Fermented Milk Product (FMP) the other acidified Milk Product (MP) for 12 weeks. H trial subjects were followed for 12 weeks, with no consumption of either product, principally to assess microbiome changes over the period. Number of individuals in different cohorts is shown in parenthesis.

**Extended Data Fig. 2: Main drivers of species enterotypes; Ordination analysis according to genus-based enterotypes; Relationship genus - species enterotypes.**

**a-d.** Normalized relative abundance of four MGS driving the enterotypes-species (ETs): *Prevotella copri* 1 (msp_0041) for ETs2, both *Bacteroides uniformis* (msp_0046) and *Bacteroides vulgatus* for ETs1, Ruminococcus sp. JC304 (msp_1643c) for ETs3. P-values were computed by pairwise Wilcoxon rank sum test. **e.** Principal coordinate analysis (PCoA) of Bray Curtis distances calculated from the weighted abundance of 1484 MGS detected in 121 individuals. The first principal plan represented 21.6% and 7.0% of whole inertia for first (horizontal) and second (vertical) axes respectively. The between class inertia was 14.8%. Genus-based "classic" enterotypes (ET) are shown. The observed probability was computed from a Monte-Carlo permutation test on the between class inertia. **f.** Overlap between ETs and ET assignment for 121 individuals of the cohort. Probability value was computed by Fisher's exact test. **g.** ET of healthy (H) and ulcerative colitis (UC) subjects. Probability was computed by a Fisher's exact test.

**Extended Data Fig. 3: Comparison of classifiers separating UC patients (n = 65) from healthy controls (n = 56).**

**a-b.** Area under the curve (AUC) values for 100 iterations on train and test data obtained with different classifiers: partial least square discriminant analysis (pls-DA), elastic net or regularized form of logistic regression (glmnet), random forests (rf), support vector machine (svm) and neural networks (nnet). 684 MGS were used as variables (Methods for details). **c-d.** Principal component analysis (PCA) computed from the median value of variable importance (varImp) for 100 repetitions with the train data for the 5 classifiers. Weighted or unweighted abundance of 684 MGS was used in panel c and d, respectively. **e.** Change of the variable importance (varImp, the median computed for 100 iterations) across different classifiers; top 100 variables for pls-DA are displayed. Yellow lines connect the same variable. VarImp is most similar for pls-DA and nnet.

**Extended Data Fig. 4: Microbiome and clinical characteristics delimiting UC subgroups.**

**a-c.** Number of MGS in healthy (H, n = 56) and ulcerative colitis cohort 1 (UCc1, n = 31) divided by the most relevant clinical categories: UC extent by Montreal classification, annual past relapse rate and fecal calprotectin cut-off values categories. p-values were computed by pairwise Wilcoxon rank sum test. **d.** Annual past relapse rate in different faecal calprotectin categories of the UCc1 cohort; p-values were computed by pairwise Wilcoxon rank sum test. **e**. Number of individuals belonging to different faecal calprotectin level groups in the three Montreal UC extent classes; p value was computed by Fisher's exact test. **f.** Enterotypes species (ETs) of H, UC cohort 2 (UCc2, n = 34) and UCc1 subjects. Probability value was obtained by a Fisher's exact test. **g.** Between-class analysis (bca) of principal component analysis (PCA) calculated from the weighted abundance table based on 1484 detected MGS in the 121 sampled individuals. The first principal plan represented 7.3% and 3.6% of whole inertia for first (horizontal) and second (vertical) axes respectively. The between class inertia percentage was 2.6%. H, UCc2 or UCc1 subjects are indicated. The probability was computed by a Monte-Carlo permutation test carried out on the between class inertia.

**Extended Data Fig. 5: Microbiome - phenotype - genotype link in aggressive UC subgroup (n = 22).**

**a-c.** Comparisons of patients from the aggressive UC (aggUC) subgroup that are both E3 and have a highRR (n = 7) and the others (n = 15): **a.** relapse rate (RR); **b**. illness duration; **c.** number of relapses. p-values were computed by Wilcoxon rank sum tests. **d.** Mutations in the PUS10 gene of individuals with relapse rate below (≤) and above (>) the median value (1.1/y.); p-values were computed by the Fisher’s exact test. **e.** Mutations in the PUS10 gene of the Montreal age at diagnosis groups (A1 < 16 y., A2 17-40 y., A3 > 40 y.; p-values were computed by the Fisher’s exact test.) **f-g.** Correlation of the fraction of the microbiome encoding CAZy glycoside hydroxylase 24 family (GH24) with the microbiome richness (Spearman's rho correlation coefficient and the associated p-value are displayed) and enterotype species (ETs) distribution (p-values were computed by pairwise Wilcoxon rank sum tests). **h-k.** Fraction of the microbiome encoding CAZy family 13 subfamily 8 (GH13_8) genes correlated with **h.** richness, **i.** enterotype species, **j.** faecal calprotectin levels and **k.** white blood cells (WBC) counts (x 10^9^/L). Spearman's rho correlation coefficients and the associated p-values are displayed; other p-values were computed by pairwise Wilcoxon rank sum tests.

**Extended Data Fig. 6: Relations of subclinical inflammation variables in UC patients.**

**a.** Faecal calprotectin in pooled UC patients (n = 65) with WBC counts below (≤) and above (>) median value (6.9 x 10^9^/L). **b-c.** WBC and fecal calprotectin in patients (n = 65) according to smoking habit. **d**. Faecal calprotectin in H cohort (n = 56) according to smoking habit. **e-f.** WBC counts and faecal calprotectin in never smoker patients (n = 27) according to Colitis Activity Index (CAI) classes (class.) asymptomatic (0) or mildly symptomatic (1-2). All p values were computed by pairwise Wilcoxon rank sum tests.

**Extended Data Fig. 7: Microbiome of UCc1 time course (TC) patients during disease activity.**

**a.** Faecal calprotectin at baseline (t0) and end time points; all patients were in remission at t0; some remained in remission (Rem, n = 12), others progressed to relapse (Rel, n = 12). Same individuals are connected by lines, p values were computed by pairwise paired or unpaired Wilcoxon tests (upper or lower values, respectively). **b.** WBC counts at baseline and end time points; same individuals are connected by lines, p values were computed by pairwise paired or unpaired Wilcoxon tests (upper or lower values, respectively). **c.** Smoking habit of UCc1 TC patients related to their baseline faecal calprotectin levels; 100 µg/g cut-off value was used. p-value was computed by the Fisher’s exact test. **d.** Smoking habit of patients related to their baseline WBC counts; p-value was computed by the Fisher’s exact test. **e.** Between-class analysis (bca) of principal component analysis (PCA) calculated from the weighted abundance table based on 1060 MGS detected in 24 UCc1 TC patients sampled at baseline. The first principal plan represented 11.0% and 8.0% of whole inertia for first (horizontal) and second (vertical) axes respectively. The between class inertia percentage was 9.0%. Baseline fecal calprotectin categorization is shown. The p-value was computed from a Monte-Carlo permutation test carried out on the between class inertia. **f.** Evolution of the number of MGS in function of the baseline fecal calprotectin levels; 100 µg/g cutoff value was used. Same individuals are connected by lines, p values were computed by pairwise paired or unpaired Wilcoxon tests (upper or lower values, respectively). **g**. Evolution of the number of MGS in function of the baseline WBC levels. Same individuals are connected by lines, p values were computed by pairwise paired or unpaired Wilcoxon tests (upper or lower values, respectively). **h**. Evolution of the number of MGS in function of the smoking status. Same individuals are connected by lines, p values were computed by pairwise paired Wilcoxon tests. **i.** End point disease activity (colitis activity index, CAI) classes (class.) as function of the smoking status; p-value was computed by the Fisher's exact test. **j.** Fraction of the microbiome encoding antibiotic resistance determinant (ARD) erythromycin methyltransferase (Erm) family genes in function of end time point calprotectin levels; p values were computed by pairwise Wilcoxon test. **k.** Fraction of the microbiome encoding Erm family genes at end point in function of the change in disease activity (Delta CAI). Spearman's rho correlation coefficient and the associated p-value are shown.

**Extended Data Fig. 8: Abundance of Fermented Milk Product strains in UCc2 trial patients and healthy individuals.**

**a-e.** Strain abundance was determined by q-PCR as described in Methods. H refers to healthy individuals (n = 9), FMP and MP to patients taking the fermented milk product (n = 17) and acidified milk (n = 14), respectively; time of sampling in weeks is indicated. The strains tested are denoted above the panels. Same individuals are connected by lines, p-values were computed by pairwise paired Wilcoxon tests.

**Extended Data Fig. 9:** **Microbiome - phenotype relations in UCc2 trial patients**

**a.** Number of MGS relative to past number of relapses, number of patients below (≤) and above (>) the median value is indicated in parentheses. p-value was computed by the Wilcoxon rank sum test. **b**. Number of past relapses for different enterotypes species (ETs); p-values were computed by pairwise Wilcoxon rank sum tests. **c.** Between-class analysis (bca) of principal component analysis (PCA) calculated from the weighted abundance table based on 1133 MGS detected in the patients. The first principal plan represented 11.1% and 7.5% of whole inertia for first (horizontal) and second (vertical) axes, respectively. The between class inertia percentage was 8%. Terciles of baseline white blood cells (WBC) counts are shown. The p-value was computed from a Monte-Carlo permutation test carried out on the between class inertia. **d-f.** Evolution of MGS numbers in function of: **d**. WBC counts; **e**. calprotectin levels; **f**. smoking habit. Same individuals are connected by lines, p values were computed by pairwise paired or unpaired Wilcoxon tests (upper or lower values, respectively).

**Extended Data Fig. 10: *Akkermansia muciniphila* detection in connection with UC phenotype and with UCc2 trial microbiome evolution**

**a-c.** Presence or absence of *Akkermansia muciniphila* (akk+ or akk-, respectively) in function of: **a**. age at diagnosis (Montreal classification); **b**. relapse rate and **c**. extra-intestinal manifestations (EIMs) of the disease for the pooled UC cohort (n = 65). **d.** Evolution of MGS numbers in function of *A. muciniphila* presence (akk+) or not (akk-) in UCc2 trial patients (n = 31). p values were computed by pairwise paired or unpaired Wilcoxon tests (upper or lower values, respectively).

**Supplementary tables**

## Supplementary Table 1: Clinical metadata according to the three initial MetaHIT (IBD UC study) cohorts

| H *vs* UCc1 *vs* UCc2 | | | | | | |
| --- | --- | --- | --- | --- | --- | --- |
| Clinical variable | H (n = 56) | UCc1 (n = 31) | UCc2 (n = 34) | p H *vs* UCc1 | p H *vs* UCc2 | p UCc1 *vs* UCc2 |
| Age (y.) | 36 [27.8-44.2] | 43 [31.5-46.5] | 45 [39.2-52] | 0.15 | **0.0011** | 0.079 |
| Gender | 26 Male (46.4%) | 9 Male (29%) | 15 Male (44.1%) | 0.17 | 1 | 0.30 |
|  | 30 Female (53.6%) | 22 Female (71%) | 19 Female (55.9%) |  |  |  |
| BMI (Kg/m²) | 24 [22-26.6] | 23.3 [20.3-26.8] | 24.2 [22.9-26.7] | 0.45 | 0.36 | 0.14 |
| Weight (Kg) | 67.5 [59-77.2] | 63 [55-71] | 68 [59.2-77.5] | 0.15 | 0.78 | 0.089 |
| Height (m) | 1.69 [1.61-1.75] | 1.63 [1.59-1.69] | 1.67 [1.58-1.74] | 0.12 | 0.51 | 0.45 |
| Smoking habit | 18 Current (32.1%) | 8 Current (25.8%) | 5 Current (14.7%) | 0.10 | **0.003** | 0.45 |
|  | 7 Former (12.5%) | 10 Former (32.3%) | 15 Former (44.1%) |  |  |  |
|  | 31 Never (55.4%) | 13 Never (41.9%) | 14 Never (41.2%) |  |  |  |
| Fecal calprotectin (µg/g) - 6 MV | 17.4 [8.59-34.5] | 64.4 [19.7-236] | 41.1 [14.6-253] | **0.00045** | **0.0031** | 0.58 |
| Age at diagnosis (y.) | - | 30 [23.5-38] | 35 [27.2-40] | - | - | 0.12 |
| Age at diagnosis (Montreal) | - | 2 A1 (6.5%) | 0 A1 (0%) | - | - | 0.48 |
|  |  | 23 A2 (74.2%) | 27 A2 (79.4%) |  |  |  |
|  |  | 6 A3 (19.4%) | 7 A3 (20.6%) |  |  |  |
| UC extent (Montreal) | - | 9 E1 (29%) | 15 E1 (44.1%) | - | - | 0.50 |
|  |  | 10 E2 (32.3%) | 9 E2 (26.5%) |  |  |  |
|  |  | 12 E3 (38.7%) | 10 E3 (29.4%) |  |  |  |
| EIMs¤ | - | 26 no (83.9%) | 26 no (76.5%) | - | - | 0.54 |
|  |  | 5 yes (16.1%) | 8 yes (23.5%) |  |  |  |
| Duration illness (mo.) | - | 90 [51-156] | 136 [56.2-187] | - | - | 0.41 |
| Treatment - 5 MV | - | 1 none (3.8%) | 3 none (8.8%) | - | - | 0.84 |
|  |  | 17 ASA (65.4%) | 20 ASA (58.8%) |  |  |  |
|  |  | 8 azathioprine (30.8%) | 11 azathioprine (32.4%) |  |  |  |
| CRP (mg/dl) - 13 MV | - | 0.24 [0.075-0.432] | 0.135 [0.095-0.242] | - | - | 0.32 |
| WBC (x10⁹/L) - 1 MV | - | 7.2 [5.55-8.8] | 6.6 [5.4-8] | - | - | 0.31 |
| Hemoglobin (gr/dl) - 1 MV | - | 13.8 [12.8-15] | 14.3 [13.2-15.1] | - | - | 0.25 |
| CAI (class.) | - | 21 0 (67.7%) | 30 0 (88.2%) | - | - | 0.069 |
|  |  | 10 1-2 (32.3%) | 4 1-2 (11.8%) |  |  |  |
| Relapses (N) | - | 7 [5.5-12] | 2 [2-4.5] | - | - | **3.9e-07** |
| Relapse rate (N/year) | - | 1.1 [0.9-1.85] | 0.3 [0.2-0.5] | - | - | **6.6e-10** |

Clinical characteristics of healthy (H) cohort, ulcerative colitis cohort 1 (UCc1) and UC cohort 2 (UCc2) subjects (n is indicated in parentheses). P-values (p) of differences (in bold if significant) were computed by Wilcoxon rank sum test or Fisher’s exact test where appropriate for group *versus* (*vs*) group comparison. Numerical variables were expressed by median followed by first - and third quartile values in square brackets. Numbers with their respective percentage in parentheses were shown if categorical. Missing values (MV), if present, expressed as total number were also reported. Extra-intestinal manifestations (EIMs¤) in 13 of 65 UC patients included: arthropathy (10/13), erythema nodosum (4/13) and uveitis (2/13) with some patients occasionally having several of them. C-reactive protein was abbreviated as CRP, white blood cells count as WBC, colitis activity index classes as CAI (class.) For details, see Methods.

## Supplementary Table 2: Clinical metadata of H and pooled UC cohorts

| H *vs* UC | | | |
| --- | --- | --- | --- |
| Clinical variable | H (n = 56) | UC (n = 65) | p |
| Age (y.) | 36 [27.8-44.2] | 43 [35-51] | **0.004** |
| Gender | 26 Male (46.4%) | 24 Male (36.9%) | 0.36 |
|  | 30 Female (53.6%) | 41 Female (63.1%) |  |
| BMI (Kg/m²) | 24 [22-26.6] | 24 [21.6-26.8] | 0.9 |
| Weight (Kg) | 67.5 [59-77.2] | 65 [57-75] | 0.51 |
| Height (m) | 1.69 [1.61-1.75] | 1.65 [1.58-1.73] | 0.18 |
| Smoking habit | 18 Current (32.1%) | 13 Current (20%) | **0.0045** |
|  | 7 Former (12.5%) | 25 Former (38.5%) |  |
|  | 31 Never (55.4%) | 27 Never (41.5%) |  |
| Fecal calprotectin (µg/g) - 6 MV | 17.4 [8.59-34.5] | 52 [14.7-245] | **9.5e-05** |

Clinical characteristics of healthy cohort (H) *versus* (*vs*) pooled ulcerative colitis (UC) cohort subjects (n indicated in parentheses.) P-values (in bold if significant) were computed Wilcoxon rank sum test or Fisher’s exact test if appropriate. Numerical variables were expressed by median followed by first - and third quartile values in square brackets. Numbers (and their percentages) were shown if categorical. If present, missing values (MV) in total numbers were also reported.

## Supplementary Table 3: Clinical categories from UC cohort 1, cohort 2 or pooled UC cohort and their impact on richness

| Richness comparison (probabilities, effect size) of H as a whole *vs* clinical categorized variables of UCc1, UCc2 or pooled UC | | | | | | | | | | |
| --- | --- | --- | --- | --- | --- | --- | --- | --- | --- | --- |
| Clinical variable | UCc1 (n = 31) | UCc2 (n = 34) | UC (n = 65) | q UCc1 | q UCc2 | q UC | p UCc1 | p UC | CliffsD Ucc1 | CliffsD UC |
| ≤ median age y. | 17 ≤ 43 y. (54.8%) | 17 ≤ 45 y. (50%) | 33 ≤ 43 y. (50.8%) | 0.09 | 0.19 | 0.057 | 0.073 | **0.037** | NC (p = 0.085) | NC (p = 0.054) |
| > median age y. | 14 > 43 y. (45.2%) | 17 > 45 y. (50%) | 32 > 43 y. (49.2%) |  |  |  |  |  | 0.32 (p = 0.069 ←) | 0.30 (p = 0.022 ←) |
| Gender^1^ | 9 Male (29%) | 15 Male (44.1%) | 24 Male (36.9%) | 0.09 | 0.19 | 0.057 | 0.075 | **0.038** | NC (p = 0.17) | NC (p = 0.079) |
|  | 22 Female (71%) | 19 Female (55.9%) | 41 Female (63.2%) |  |  |  |  |  | 0.30 (p = 0.042 ←) | 0.28 (p = 0.018 ←) |
| < 25 Kg/m² BMI cutoff | 19 < 25 Kg/m² (61.3%) | 19 > 25 Kg/m² (55.9%) | 38 < 25 Kg/m² (58.5%) | 0.09 | 0.19 | 0.057 | **0.041** | **0.025** | 0.38 (p = 0.013 ←) | 0.32 (p = 0.0088 ←) |
| > 25 Kg/m² BMI cutoff | 12 > 25 Kg/m² (38.7%) | 15 > 25 Kg/m² (44.1%) | 27 > 25 Kg/m² (41.5%) |  |  |  |  |  | NC (p = 0.40) | NC (p = 0.14) |
| ≤ median weight Kg | 18 ≤ 63 Kg (58.1%) | 17 ≤ 68 Kg (50%) | 35 ≤ 65 Kg (53.8%) | 0.09 | 0.19 | 0.057 | 0.053 | **0.015** | 0.37 (p = 0.021 ←) | 0.37 (p = 0.0035 ←) |
| > median weight Kg | 13 > 63 Kg (41.9%) | 17 > 68 Kg (50%) | 30 > 65 Kg (46.2%) |  |  |  |  |  | NC (p = 0.27) | NC (p = 0.23) |
| ≤ median height m | 18 ≤ 1.63 m (58.1%) | 18 ≤ 1.67 m (52.9%) | 36 ≤ 1.65 m (55.4%) | 0.09 | 0.19 | 0.057 | 0.074 | **0.033** | 0.29 (p = 0.07 ←) | NC (p = 0.052) |
| > median height m | 13 > 1.63 m (41.9%) | 16 > 1.67 m (47.1%) | 29 > 1.65 m (44.6%) |  |  |  |  |  | NC (p = 0.086) | 0.31 (p = 0.032 ←) |
| Smoking habit^1^ | 8 Current (25.8%) | 5 Current (14.7%) | 13 Current (20%) |  |  |  |  |  |  | NC (p = 0.23) |
|  | 10 Former (32.3%) | 15 Former (44.1%) | 25 Former (38.5%) | 0.12 | 0.25 | 0.066 |  | 0.055 |  | 0.36 (p = 0.011 ←) |
|  | 13 Never (41.9%) | 14 Never (41.2%) | 27 Never 41.5%) |  |  |  |  |  |  | NC (p = 0.12) |
| < 50 µg/g Fecal calprotectin cutoff | 13 < 50 µg/g (44.8%) | 18 < 50 µg/g (54.5%) | 31 < 50 µg/g (50%) |  |  |  |  |  | NC (p = 0.11) | 0.29 (p = 0.027 ←) |
| [50,250] Fecal calprotectin cutoff | 10 [50,250] (34.5%) | 6 [50,250] (18.2%) | 16 [50,250] (25.8%) | 0.09 | 0.19 | 0.084 | 0.057 | 0.079 | 0.49 (p = 0.014 ←) | NC (p = 0.081) |
| > 250 µg/g Fecal calprotectin cutoff | 6 > 250 µg/g (20.7%) – 2 MV | 9 > 250 µg/g (27.3%) – 1 MV | 15 > 250 µg/g (24.2%) – 3 MV |  |  |  |  |  | NC (p = 0.85) | NC (p = 0.17) |
| Age at diagnosis (Montreal)^1^ | 2 A1 (6.5%) | 0 A1 (0%) | 2 A1 (3.1%) |  |  |  |  |  |  | NC (p = 0.44) |
|  | 23 A2 (74.2%) | 27 A2 (79.4%) | 50 A2 (76.9%) | 0.12 | 0.19 | 0.066 |  | **0.054** |  | NC (p = 0.048 ←) |
|  | 6 A3 (19.4%) | 7 A3 (20.6%) | 13 A3 (20%) |  |  |  |  |  |  | 0.44 (p = 0.014) |
| UC extent (Montreal)^1^ | 9 E1 (29%) | 15 E1 (44.1%) | 24 E1 (36.9%) |  |  |  |  |  | NC (p = 0.44) | NC (p = 0.092) |
|  | 10 E2 (32.3%) | 9 E2 (26.5%) | 19 E2 (29.2%) | 0.09 | 0.29 | 0.063 | 0.050 | **0.045** | NC (p = 0.36) | NC (p = 0.24) |
|  | 12 E3 (38.7%) | 10 E3 (29.4%) | 22 E3 (33.8%) |  |  |  |  |  | 0.49 (p = 0.0085 ←) | 0.38 (p = 0.0095 ←) |
| EIMs^1^ | 26 no (83.9%) | 26 no (76.5%) | 52 no (80%) | 0.09 | 0.19 | 0.057 | 0.074 | **0.037** | 0.29 (p = 0.036 ←) | 0.26 (p = 0.019 ←) |
|  | 5 yes (16.1%) | 8 yes (23.5%) | 13 yes (20%) |  |  |  |  |  | NC (p = 0.23) | NC (p = 0.096) |
| ≤ median duration illness mo. | 16 ≤ 90 mo. (51.6%) | 17 ≤ 136 mo. (50%) | 33 ≤ 120 mo. (50.8%) | 0.09 | 0.19 | 0.057 | 0.064 | **0.038** | 0.35 (p = 0.035 ←) | NC (p = 0.037) |
| > median duration illness mo. | 15 > 90 mo. (48.4%) | 17 > 136 mo. (50%) | 32 > 120 mo. (49.2%) |  |  |  |  |  | NC (p = 0.16) | 0.27 (p = 0.034 ←) |
| Treatment^1^ | 1 none (3.8%) | 3 none (8.8%) | 4 none (6.7%) |  |  |  |  |  | NC (p = 0.17) | NC (p = 0.22) |
|  | 17 ASA (65.4%) | 20 ASA (58.8%) | 37 ASA (61.7%) | 0.09 | 0.19 | 0.066 | **0.046** | 0.059 | 0.39 (p = 0.016 ←) | 0.32 (p = 0.0093 ←) |
|  | 8 azathioprine (30.8%) – 5 MV | 11 azathioprine (32.4%) | 19 azathioprine (31.7%) – 5 MV |  |  |  |  |  | NC (p = 0.43) | NC (p = 0.46) |
| ≤ median CRP mg/dl | 12 ≤ 0.24 mg/dl (50%) | 14 ≤ 0.14 mg/dl (50%) | 26 ≤ 0.17 mg/dl (50%) | 0.12 | 0.36 | 0.110 | ND |  |  |  |
| > median CRP mg/dl | 12 > 0.24 mg/dl (50%) – 7 MV | 14 > 0.14 mg/dl (50%) – 6 MV | 26 > 0.17 mg/dl (50%) – 13 MV |  |  |  |  |  |  |  |
| ≤ median WBC x10⁹/L | 17 ≤ 7.2 x10⁹/L (57.6%) | 19 ≤ 6.6 x10⁹/L (57.6%) | 33 ≤ 6.9 x10⁹/L (51.6%) | 0.09 | 0.19 | 0.057 | 0.055 | **0.010** | 0.36 (p = 0.024 ←) | 0.38 (p = 0.0033 ←) |
| > median WBC x10⁹/L | 14 > 7.2 x10⁹/L (45.2%) | 14 > 6.6 x10⁹/L (42.4%) – 1 MV | 31 > 6.9 x10⁹/L (48.4%) – 1 MV |  |  |  |  |  | NC (p = 0.22) | NC (p = 0.28) |
| ≤ median hemoglobin gr/dl | 16 ≤ (51.6%) | 17 ≤ (51.5%) | 33 ≤ (51.6%) | 0.09 | 0.24 | 0.057 | 0.075 | **0.035** | 0.30 (p = 0.072 ←) | 0.32 (p = 0.013 ←) |
| > median hemoglobin gr/dl | 15 > 13.8 gr/dl (48.4%) | 16 > 14.3 gr/dl (48.5%) – 1 MV | 31 > 14.1 gr/dl (48.4%) – 1 MV |  |  |  |  |  | NC (p = 0.083) | NC (p = 0.12) |
| CAI (class.)^1^ | 21 0 (67.7%) | 30 0 (88.2%) | 51 0 (78.5%) | 0.09 | 0.19 | 0.057 | 0.046 | **0.038** | 0.36 (p = 0.015 ←) | 0.26 (p = 0.02 ←) |
|  | 10 1-2 (32.3%) | 4 1-2 (11.8%) | 14 1-2 (21.5%) |  |  |  |  |  | NC (p = 0.45) | NC (p = 0.087) |
| ≤ median N relapses | 17 ≤ 7 relapses (54.8%) | 21 ≤ 2 relapses (61.8%) | 37 ≤ 5 relapses (56.9%) | 0.09 | 0.19 | 0.057 | 0.066 | **0.038** | NC (p = 0.15) | 0.26 (p = 0.032 ←) |
| > median N relapses | 14 > 7 relapses (45.2%) | 13 > 2 relapses (38.2%) | 28 > 5 relapses (43.1%) |  |  |  |  |  | 0.37 (p = 0.034 ←) | NC (p = 0.039) |
| ≤ median relapse rate N/year | 16 ≤ 1.1/year (51.6%) | 20 ≤ 0.3/year (58.8%) | 33 ≤ 0.5/year (50.8%) | 0.09 | 0.19 | 0.057 | **0.024** | **0.013** | NC (p = 0.37) | NC (p = 0.22) |
| > median relapse rate N/year | 15 > 1.1/year (48.4%) | 14 > 0.3/year (41.2%) | 32 > 0.5/year (49.2%) |  |  |  |  |  | 0.45 (p = 0.0074 ←) | 0.39 (p = 0.0027 ←) |

Richness (MGS N) comparison of (un-split) healthy cohort (H, n = 56) *versus* (*vs*) ulcerative colitis cohort 1 (UCc1), *vs* UC cohort 2 (UCc2), *vs* pooled (UC) cohort (n indicated in parentheses) through their clinical categories. Variables are all categorized, either directly extracted from Supplementary table 1 (^1^) if originally categorical, or discretized, if numerical, based on their median (inferior or equal ≤; strictly superior >) or on cut-off (strictly inferior <; >) values for each patient cohort (clinical metadata mining detailed in Methods). Median value or cutoff (BMI < or > 25 kg/m²; Fecal calprotectin < 50 or [50,250] or > 250 µg/g) or original categories, cohort by cohort, are surrounded by their respective numbers and percentages (in parentheses). Missing values (MV) are also reported. For each clinical categorized variable, the order of their categories, so-called modalities, are displayed row by row (ordered top to bottom): first or last modality if k = 2, mid or intermediate modality between if k = 3. Confronted to those of non-split H cohort as a whole, MGS N was compared for each category for the three patient cohorts. Kruskal-Wallis rank sum test was computed and false discovery rate detection was applied (Benjamini-Hochberg method) with q-values indicated for the three (q UCc1, q UCc2 and q UC columns). If q-value are < 0.10, corresponding p–values (p UCc1 and p UC columns) are shown (in bold if significant), if not, cells are left empty the p UCc2 column lacking. Therefore, in UCc1 and pooled UC only, effect size or Cliff’s Delta (CliffsD) was calculated for the category in relation to the lowest probability (← annotation in parentheses) resulted from (with H) two by two Wilcoxon rank sum test (p in parentheses). Otherwise, CliffsD are not calculated (NC) or are empty cells if they correspond to other empty cells. EIMs notified extra-intestinal manifestations (for details see also Supplementary Table 1). CRP denoted C-reactive protein, WBC white blood cells count, CAI (class.) colitis activity index classes.

## Supplementary Table 4: Clinical metadata of UC cohort 2 trial (n = 31)

| Allocation Probiotic *vs* placebo (UCc2 trial at baseline or 0w) | | | |
| --- | --- | --- | --- |
| Clinical variable | FMP arm (n = 17) | MP arm (n = 14) | p |
| Age (y.) | 41 [39-54] | 47.5 [43.2-51.5] | 0.5 |
| Gender | 7 Male (41.2%) | 8 Male (57.1%) | 0.48 |
|  | 10 Female (58.8%) | 6 Female (42.9%) |  |
| BMI (Kg/m²) | 24 [22.7-28.8] | 24.8 [23.5-26] | 0.77 |
| Weight (Kg) | 70 [60-71] | 72.5 [65.2-80.8] | 0.59 |
| Height (m) | 1.61 [1.58-1.75] | 1.69 [1.67-1.74] | 0.2 |
| Smoking habit | 2 Current (11.8%) | 3 Current (21.4%) | 0.79 |
|  | 8 Former (47.1%) | 6 Former (42.9%) |  |
|  | 7 Never (41.2%) | 5 Never (35.7%) |  |
| Fecal calprotectin (µg/g) - 1 MV | 76.4 [16.8-479] | 21.2 [9.6-29.8] | 0.065 |
| Age at diagnosis (y.) | 32 [27-39] | 38 [29.8-44] | 0.29 |
| Age at diagnosis (Montreal) | 15 A2 (88.2%) | 10 A2 (71.4%) | 0.37 |
|  | 2 A3 (11.8%) | 4 A3 (28.6%) |  |
| UC extent (Montreal) | 10 E1 (58.8%) | 2 E1 (14.3%) | **0.016** |
|  | 2 E2 (11.8%) | 7 E2 (50%) |  |
|  | 5 E3 (29.4%) | 5 E3 (35.7%) |  |
| EIMs | 13 no (76.5%) | 11 no (78.6%) | 1 |
|  | 4 yes (23.5%) | 3 yes (21.4%) |  |
| Duration illness (mo.) | 110 [52-204] | 148 [69.8-187] | 0.71 |
| Treatment | 2 none (11.8%) | 1 none (7.1%) | 0.87 |
|  | 10 ASA (58.8%) | 7 ASA (50%) |  |
|  | 5 azathioprine (29.4%) | 6 azathioprine (42.9%) |  |
| CRP (mg/dl) - 5 MV | 0.13 [0.08-0.39] | 0.15 [0.12-0.24] | 0.63 |
| WBC (x10⁹/L) - 1 MV | 6.6 [5.8-8] | 6.1 [5.4-7] | 0.57 |
| Hemoglobin (gr/dl) - 1 MV | 14.1 [13.2-14.7] | 15 [14.1-15.6] | 0.14 |
| CAI (class.) | 14 0 (82.4%) | 14 0 (100%) | 0.23 |
|  | 3 1-2 (17.6%) | 0 1-2 (0%) |  |
| Relapses (N) | 2 [2-5] | 2.5 [1.25-4.5] | 0.95 |
| Relapse rate (N/year) | 0.3 [0.2-0.5] | 0.3 [0.2-0.5] | 0.51 |

Clinical characteristics of UC cohort 2 (UCc2) trial subjects at week 0 (0w) or baseline according to fermented milk product (FMP) or acidified milk product (MP) consumption group assignment (n is indicated in parentheses). P-values (p) of differences (in bold if significant) were computed by Wilcoxon rank sum test or Fisher’s exact test where appropriate for group *versus* (*vs*) group comparison. Numerical variables were expressed as median followed by first - and third quartile values in square brackets. Numbers with their respective percentage in parentheses were shown if categorical. Missing values (MV), if present, expressed as total number were also reported. EIMs notified extra-intestinal manifestations (for details see Supplementary Table 1), CRP denoted C-reactive protein, WBC white blood cells count, CAI (class.) colitis activity index classes.

## Supplementary Table 5: Metadata of UC cohort 2 trial both akk- and fecal calprotectin < 250 µg/g (n = 17)

| Allocation FMP *vs* MP (UCc2 trial patients, both akk- and calprotectin < 250 µg/gr at baseline or 0w) | | | |
| --- | --- | --- | --- |
| Clinical variable | FMP arm (n = 9) | MP arm (n = 8) | p |
| Age (y.) | 51 [40-55] | 47.5 [43.5-50.5] | 0.89 |
| Gender | 2 Male (22.2%) | 5 Male (62.5%) | 0.15 |
|  | 7 Female (77.8%) | 3 Female (37.5%) |  |
| BMI (Kg/m²) | 24 [21.9-28.5] | 24.7 [23.4-25.5] | 0.67 |
| Weight (Kg) | 60 [59-70] | 72.5 [64.2-80.2] | 0.36 |
| Height (m) | 1.58 [1.57-1.65] | 1.72 [1.69-1.75] | **0.048** |
| Smoking habit | 1 Current (11.1%) | 1 Current (12.5%) | 0.8 |
|  | 3 Former (33.3%) | 4 Former (50%) |  |
|  | 5 Never (55.6%) | 3 Never (37.5%) |  |
| Fecal calprotectin (µg/g) | 27.5 [14.6-76.4] | 13.8 [9.22-21.8] | 0.17 |
| Age at diagnosis (y.) | 35 [27-40] | 38 [33.5-46.8] | 0.41 |
| Age at diagnosis (Montreal) | 7 A2 (77.8%) | 5 A2 (62.5%) | 0.37 |
|  | 2 A3 (22.2%) | 3 A3 (37.5%) |  |
| UC extent (Montreal) | 5 E1 (55.6%) | 1 E1 (12.5%) | 0.23 |
|  | 2 E2 (22.2%) | 4 E2 (50%) |  |
|  | 2 E3 (22.2%) | 3 E3 (37.5%) |  |
| EIMs | 8 no (88.9%) | 6 no (75%) | 0.58 |
|  | 1 yes (11.1%) | 2 yes (25%) |  |
| Duration illness (mo.) | 110 [52-240] | 111 [33.8-160] | 0.83 |
| Treatment | 1 none (11.1%) | 1 none (12.5%) | 1 |
|  | 5 ASA (55.6%) | 5 ASA (62.5%) |  |
|  | 3 azathioprine (33.3%) | 2 azathioprine (25%) |  |
| CRP (mg/dl) - 1 MV | 0.115 [0.0725-0.39] | 0.165 [0.115-0.225] | 0.6 |
| WBC (x10⁹/L) | 6.9 [5.6-8] | 6.5 [5.49-7.52] | 0.74 |
| Hemoglobin (gr/dl) | 14.1 [13.4-14.6] | 15 [13.3-15.5] | 0.39 |
| CAI (class.) | 8 0 (88.9%) | 8 0 (100%) | 0.48 |
|  | 1 1-2 (11.1%) | 0 1-2 (0%) |  |
| Relapses (N) | 2 [2-8] | 1.5 [1-3] | 0.34 |
| Relapse rate (N/year) | 0.4 [0.2-0.5] | 0.35 [0.175-0.5] | 0.52 |

Comparison of clinical characteristics of UC cohort 2 (UCc2) trial subjects, lacking *Akkermansia muciniphila* (akk-) and having fecal calprotectin < 250 µg/g at week 0 (0w), according to fermented milk product (FMP) or acidified milk product (MP) consumption group assignment (n is indicated in parentheses). P-values (p) of differences (in bold if significant) were computed by Wilcoxon rank sum test or Fisher’s exact where appropriate for group *versus* (*vs*) comparison. Numerical variables were expressed as median followed by first - and third quartile values in square brackets. Numbers with their respective percentage in parentheses were shown if categorical. Missing values (MV), if present, expressed as total number were also reported. EIMs notified extra-intestinal manifestations (for details see Supplementary Table 1), CRP denoted C-reactive protein, WBC white blood cells count, CAI (class.) colitis activity index classes.
